# From Somatic Driver to Molecular Therapy in Retinal, Choroidal and Periocular Vascular Disease: Mosaicism, Targetable Pathways and Ocular Toxicity

**DOI:** 10.3390/ijms27188244

**Published:** 2026-09-16

**Authors:** Wojciech Adamski

**Affiliations:** 1 Department of Ophthalmology, The Regional Hospital in Poznan, The Greater Poland Specialist Center, 60-479 Poznan, Poland; wojciech.adamski@lutycka.pl; 2 Laboratory of Vision Science and Optometry, Faculty of Physics and Astronomy, Adam Mickiewicz University, 61-614 Poznan, Poland

**Keywords:** vascular anomalies, somatic mosaicism, *GNAQ*, *PIK3CA*, *GJA4*, retinal hemangioblastoma, choroidal hemangioma, targeted therapy, belzutifan, MEK inhibitor-associated retinopathy

## Abstract

The identification of somatic drivers has turned a field once based on morphology into a set of molecular pathways that can potentially be targeted with drugs. Vascular lesions of the eye and ocular adnexa used to be classified by their appearance; many of them are now defined by a post-zygotic variant in a signaling cascade for which an inhibitor is already available. This review groups these lesions by signaling axis and examines how close each pathway is to clinical application. Belzutifan seems to be the most advanced. In a single-arm phase 2 subgroup with prospectively graded retinal endpoints, HIF-2α inhibition improved all 16 evaluable eyes in 12 participants, without a comparator arm. PI3K and mTOR inhibition is supported by clinical studies in predominantly non-ocular venous and lymphatic malformations, with more consistent evidence for symptomatic benefit than for volume reduction. Direct orbital evidence remains limited to small series. MEK inhibition treats RAS-driven disease in other locations, but it can also cause a retinopathy of its own, which makes it both a therapy and an ophthalmic concern. Gq/11 signaling accounts for most capillary malformations, Sturge–Weber syndrome and both choroidal hemangiomas, which are separated by the mutated codon rather than by histology, and it remains a preclinical target. Junctional *GJA4* signaling defines the orbital cavernous venous malformation, probably the clearest example of a disease defined by its driver. Genotyping of ocular lesions is limited by tissue access and by variant allele frequencies that are commonly missed by standard panels.

## 1. Introduction

The terminology of ocular and periocular vascular lesions long preceded their molecular explanation. The suffix “-angioma” was applied to many red or vascular-appearing lesions, even when those entities differed in age of onset, anatomical site, growth behavior and biology. As a result, the same name came to cover a periocular tumor of infancy, a static intraconal orbital mass in an adult, an orange posterior-pole choroidal lesion and a peripheral retinal nodule with a feeder vessel. The shared label also implied, often incorrectly, that each was a proliferating vascular tumor.

Order was brought to this terminology by the International Society for the Study of Vascular Anomalies (ISSVA), which separates vascular tumors, in which endothelial cells proliferate, from vascular malformations, developmental errors with largely quiescent endothelium [1]. This division was a substantial advance and it remains the working clinical framework. However, it was drawn from histology and clinical behavior, and the decade that followed has tested it against genotype.

This review examines how these molecular findings modify the traditional clinicopathological classification of ocular vascular lesions. Somatic, post-zygotic variants are now established as drivers of most vascular anomalies. Such a variant arises in a single cell after fertilization, and only the descendants of that cell carry it. The mapping between a driver gene and a morphological name is not one-to-one, and it does not respect the tumor-malformation boundary.

*GNAQ* alone can drive four different lesions: a capillary malformation of the face, a diffuse choroidal lesion, a circumscribed choroidal lesion, and a congenital hemangioma classified as a tumor. Their phenotype appears to depend on the affected codon, the cell lineage carrying the variant and, probably, the developmental timing of the somatic event.

The mass that orbital surgeons have long called cavernous hemangioma carries a recurrent variant in *GJA4*, a gap-junction gene, and its proliferative index is close to zero. It therefore shares its genotype with cutaneous and hepatic venous malformations. ISSVA has responded by annotating its classification with causative genes. What is more, it has been stated that the document is constantly developing [2,3]. The field itself describes this transition as a move from a clinicohistologic to a genetic framework [4,5,6].

The eye and its surrounding tissues are especially important in this discussion. Many of these lesions can be seen directly, repeatedly and at high magnification. Their response to treatment can therefore be measured with imaging rather than by estimating volume. The retina and choroid are particularly suitable for longitudinal assessment because vascular lesions can be examined directly and repeatedly with high-resolution imaging. The orbit is different: the lesion lies in a closed bony space next to the optic nerve, so even limited enlargement can matter. For periocular infantile hemangioma, the key outcome is not lesion size but the effect on refraction and vision as it may impede visual development.

The molecular understanding has also brought new drugs, and some of them can themselves damage the tissues of the eye. Mitogen-activated protein kinase kinase (MEK) inhibitor-associated retinopathy is a predictable consequence of blocking the pathway that drives arteriovenous malformation. These agents are often prescribed by dermatologists and pediatricians, but it is the ophthalmologists who have to recognize and monitor the retinal toxicity.

For this reason, this review is organized by signaling axis rather than by anatomy or ISSVA category. Section 2 describes the literature search and the selection criteria. Section 3 compares the two classification frameworks with each other. Section 4, Section 5, Section 6 and Section 7 follow the three cytoplasmic cascades that account for most of these lesions, namely Gq/11 signaling, the PI3K/AKT/mTOR axis with its receptor-proximal partner *TEK*/TIE2, and RAS/MAPK, together with the points at which they converge. Section 8 and Section 9 describe two mechanisms that are not cascades in the same sense, although each of them defines an important ophthalmic entity. Endothelial junctional signaling explains the orbital cavernous venous malformation, the clearest case in this review of a name that has outlived its biology. Hypoxia signaling explains the von Hippel–Lindau (*VHL*)-driven retinal hemangioblastoma, the only lesion discussed here for which a systemic drug has an ocular endpoint, as well as the hypoxic program of infantile hemangioma.

Section 10 then asks what determines the phenotype when the driver gene is constant. Section 11 turns to practice and covers how a variant present at a low variant allele frequency (VAF), the proportion of DNA copies in a sample that carry the variant, can be detected in a small periocular specimen; Section 12 and Section 13 address targeted therapy and future directions. Some lesions, including orbital varix and conjunctival malformations, have no known molecular basis and are identified as such, rather than being assigned to the nearest characterized entity.

In practice, the clinician still starts with the phenotype, because that is what brings the patient to care. The difficulty is that older lesion names often imply a pattern of proliferation, natural history or treatment response that the genotype does not support.

## 2. Literature Search and Selection

This is a narrative review; no formal systematic-review protocol was registered, and no PRISMA flow diagram is reported. The literature was identified by searching PubMed/MEDLINE (NCBI Entrez) from database inception to 26 July 2026, supplemented by backward and forward citation tracking of all included primary studies and by screening of the reference lists of recent reviews. ClinicalTrials.gov and the EU Clinical Trials Register were searched for registered trials of the agents discussed, and the websites of the U.S. Food and Drug Administration and the European Medicines Agency were consulted for the regulatory status of belzutifan, alpelisib, sirolimus and MEK inhibitors.

Search strategies combined terms for the lesion (e.g., “choroidal hemangioma”, “retinal hemangioblastoma”, “cavernous venous malformation of the orbit”, “racemose hemangioma”, “vascular malformation”, “infantile hemangioma”, “Sturge-Weber”), terms for the molecular event (e.g., “somatic mutation”, “mosaic”, “variant allele frequency”, “*GNAQ*”, “*GNA11*”, “*PIK3CA*”, “*TEK*”, “*KRAS*”, “*MAP2K1*”, “*GJA4*”, “*VHL*”, “HIF-2α”), and terms for therapy or toxicity (e.g., “targeted therapy”, “sirolimus”, “alpelisib”, “belzutifan”, “trametinib”, “MEK inhibitor”, “retinopathy”, “ocular adverse event”). No language restriction was applied at the search stage; reports were assessed in full text only when an English-language version was available. During revision, a focused supplementary web search of indexed literature and publisher pages on 5 September 2026 examined orbital lymphatic malformation with *PIK3CA* or alpelisib, and vascular malformation with CRISPR, base editing, prime editing, DNA correction, antisense, siRNA, RNA interference or CasRx, including combinations with *PIK3CA*, *TEK* and *KRAS*. Relevant primary reports were checked in full text; this targeted update was not a repeat of the original database screening.

Searching was targeted rather than exhaustive: queries were narrowed iteratively as the relevant driver genes, agents and toxicity syndromes emerged, and records were selected by title and abstract for full-text assessment. Approximately 450 reports were assessed in full text. Screening, eligibility assessment and evidence ranking were performed by the single author, without duplicate independent screening or adjudication by a second reviewer, and no screening log was kept, so record-level counts at each stage are not reported. Eligibility was judged against three criteria: (i) the report concerned a vascular lesion of the retina, choroid, orbit or ocular adnexa, or a multisystem vascular anomaly with a documented ocular or periocular phenotype; non-ocular studies were also eligible when they directly informed a shared mechanism, molecular diagnostic method or therapeutic extrapolation discussed here; (ii) it contributed molecular data (somatic or germline variant identification, mechanistic or reporter-assay work), diagnostic data on mosaic-variant detection, or treatment and outcome data for a pathway-targeted agent or its ocular toxicity; and (iii) primary clinical and experimental studies were prioritized. Systematic reviews, meta-analyses, consensus statements and classification documents were used for synthesis or contextual recommendations and to locate primary studies. Trial registries and regulatory documents informed trial status and authorized indications. Relevant preprints were eligible as explicitly labeled preliminary mechanistic evidence, rather than as established clinical evidence. Lesions were excluded when no plausible molecular or therapeutic link to the ocular or periocular region exists; on this basis PHACE syndrome, in which no somatic driver has been identified and no molecular data from ocular or periocular tissue are available, and hereditary hemorrhagic telangiectasia, which is a germline monogenic disorder rather than a somatic mosaic one, are not treated in dedicated sections and are mentioned only where they intersect the pathways discussed. Infantile hemangioma is retained despite the absence of an identified somatic driver, because it is the commonest periocular vascular tumor of childhood and both its pathway-directed treatment and its ocular effects are discussed here.

Treatment evidence is described using the design-based tiers defined in Section 12, with the anatomical source of the evidence stated separately. These tiers are descriptive, not a validated certainty-of-evidence assessment, and do not by themselves establish therapeutic readiness. Molecular evidence is assessed separately from treatment evidence. Where a finding was not identified, the statement is restricted to the stated search scope and modality; DNA correction, antisense or siRNA treatment, and RNA-targeting CRISPR are distinguished in Section 13.2. A negative literature search is not evidence that an intervention has failed experimentally. Genotype–phenotype statements retain lesion-specific denominators because the strength of these associations differs markedly between conditions.

## 3. Classification Frameworks: Phenotype Meets Genotype

The ISSVA system divides vascular anomalies into tumors and malformations. Tumors are subclassified as benign, locally aggressive or borderline, and malignant, whereas malformations are named after the vessels involved—capillary, venous, lymphatic or arteriovenous—in simple, combined and syndrome-associated forms [1].

This division is clinically useful. It correctly predicts that antiproliferative therapy may affect a proliferating tumor but has little to act on in a malformation, and it separates lesions that involute from those that grow together with the patient. Later revisions added a molecular annex that places causative genes next to the morphological categories, and the classification is now maintained as an expanding document open to revision [2,3].

Adding the molecular axis to this scheme reveals three recurring conflicts. First, one gene can cross the tumor-malformation boundary: activating variants in *GNAQ* and *GNA11* at codon 209 cause congenital hemangioma, an ISSVA tumor, whereas variants at codon 183 in the same genes cause capillary malformation, an ISSVA malformation. Section 4.3 explains why these two codons behave differently.

In the second, one morphological category contains several unrelated genotypes. Lesions grouped as slow-flow venous malformations divide into *TEK*-driven and *PIK3CA*-driven disease, and the two are largely mutually exclusive. The orbital cavernous lesion falls into the same broad category, yet it is driven by neither of these genes. The third discrepancy concerns the eye most directly, because one name covers two different diseases. The diffuse and the circumscribed choroidal hemangioma differ by codon, and therefore also in natural history and in response to therapy; in most cases they also differ in prognosis.

Because the variant arises post-zygotically, the patient is a mosaic of mutant and normal cells, and how much of the body carries it depends on how early in development it appeared.

Happle’s paradominant model describes exactly this arrangement: an allele that would be lethal in every cell can survive in a mosaic form. This model has now been demonstrated directly for *GNAQ* p.Arg183Gln in a mouse model, and Section 10 presents that experiment together with what it implies about developmental timing [7].

This has two practical consequences. First of all, segmental and patterned distributions, which the older literature treated as descriptive curiosities, are in fact the visible imprint of a clone. Secondly, peripheral blood is also the wrong material for diagnosis, because the informative allele is generally absent from it.

Sequencing depth adds another constraint, discussed in detail in Section 11. The key point is that a negative result often reflects the assay or the specimen, not the absence of a driver. Published genotype–phenotype links are therefore biased toward lesions that are large enough to biopsy, accessible enough to sample and common enough to have attracted study.

These associations therefore differ in strength. A formal review found that many reported genotype–phenotype links in vascular anomalies have limited or no supporting evidence [8], and access to testing varies between centers [9]. The genotype axis is thus incomplete—dense in some areas, sparse in others, and thinnest in the four periocular entities named in Section 10.

**Figure 1:**
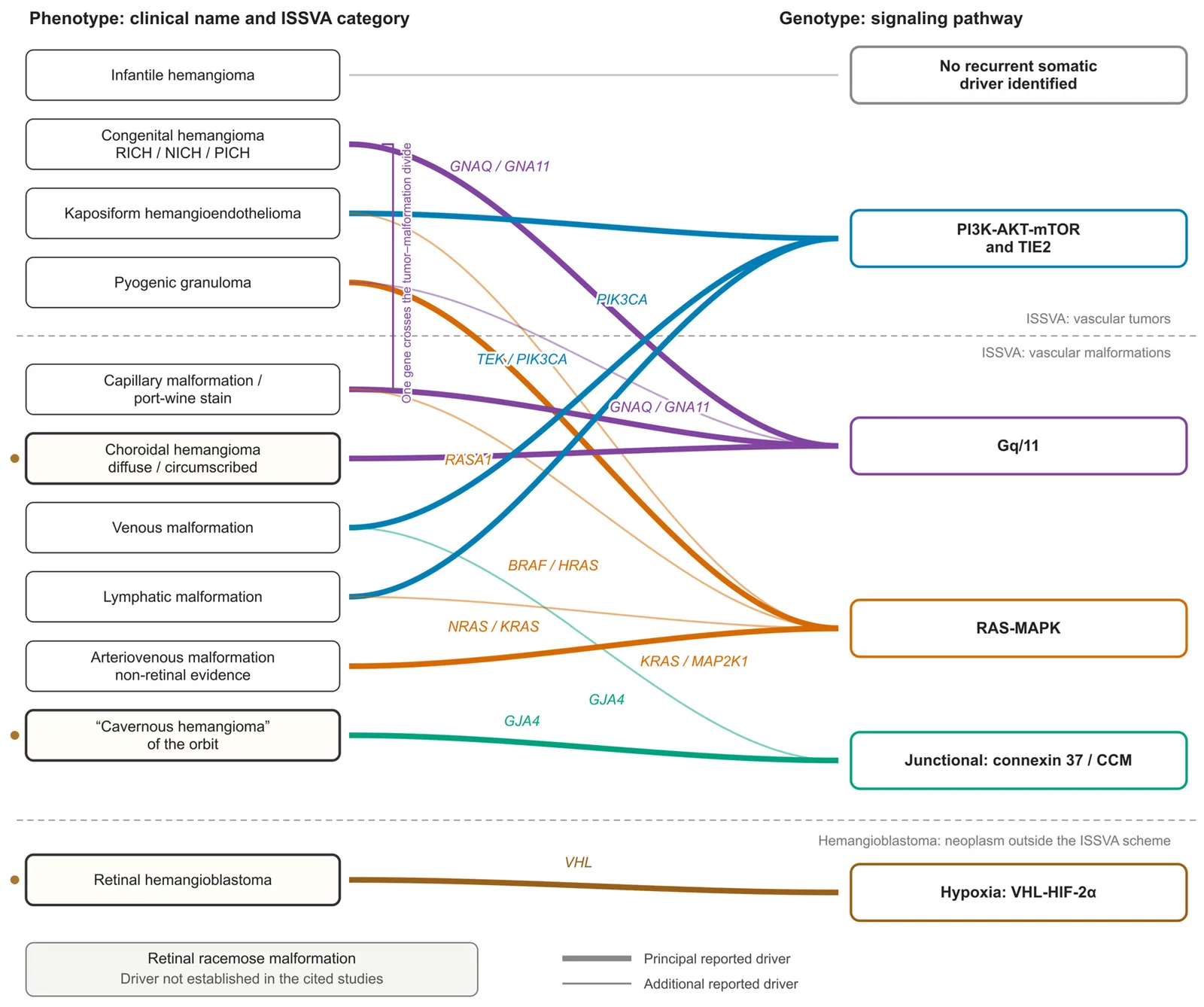
The phenotype axis and the genotype axis of periocular and orbital vascular anomalies map onto each other many-to-many, and the mapping crosses the tumor/malformation divide on which the ISSVA (International Society for the Study of Vascular Anomalies) classification is built. Left column, lesion categories as they are named clinically and histologically, separated by the upper dashed rule into ISSVA vascular tumors and vascular malformations. Retinal hemangioblastoma is set below a second dashed rule because it is a true neoplasm and falls outside the ISSVA scheme altogether, which classifies vascular tumors and malformations rather than the hemangioblastic neoplasms of von Hippel–Lindau disease. Right column, the molecular axes: Gq/11, PI3K-AKT-mTOR together with the endothelial receptor tyrosine kinase TIE2, RAS-MAPK, the junctional axis comprising connexin 37 and the cerebral cavernous malformation (CCM) complex, and the hypoxia axis comprising VHL and HIF-2α. Ribbon thickness distinguishes the principal reported driver of a lesion category from additional reported drivers, and ribbon color carries the pathway axis; the same colors are used for the same axes in Figures 2–5. Two crossings are marked. First, *GNAQ* appears on both sides of the ISSVA divide, at codon 209 in congenital hemangioma and at codon 183 in capillary malformation and Sturge–Weber syndrome [10,11,12]. Second, the orbital lesion conventionally called cavernous hemangioma carries a somatic *GJA4* variant and sits in the junctional axis rather than anywhere near the hemangiomas, with no *BRAF* or *KRAS* variant found on targeted testing and no *GNAQ* or *GNA11* variant reported in a three-case focused-exome screen [13,14]. Infantile hemangioma is placed against “no recurrent somatic driver identified” because its mechanism is described in terms of receptor imbalance and a placental-type endothelial signature rather than a recurrent somatic driver gene [15,16,17]. Abbreviations: ISSVA, International Society for the Study of Vascular Anomalies; RICH, NICH and PICH, rapidly involuting, non-involuting and partially involuting congenital hemangioma; CCM, cerebral cavernous malformation; VHL, von Hippel–Lindau; HIF, hypoxia-inducible factor. Lesions of the retina, choroid or orbit are marked with a filled circle and a heavier box outline; these are the entities on which this review concentrates. The RAS-MAPK connection represents evidence from non-retinal arteriovenous malformations. Retinal racemose malformation is shown separately because its driver is not established by the cited studies. *PIK3CA* findings in CVMS concern orbital lymphatic and lymphaticovenous malformations, as discussed in Section 5.2. The pale fill carries the same meaning as the circle and the heavier outline. The thin grey line from infantile hemangioma is a connector to the absence of a recurrent driver, not a driver ribbon.

The position taken in this review is specific: morphology remains the entry point and the ISSVA categories remain the shared vocabulary, but where a genotype is known, it should be treated as the more reliable predictor of biological behavior and of drug response. Figure 1 sets the clinical categories against the molecular axes and marks the two points at which they cross.

## 4. The Gq/11 Axis

### 4.1. One Codon Under Many Names

Facial capillary malformation was among the first vascular anomalies in which a recurrent somatic driver was defined. The key variant, *GNAQ* c.548G>A, p.Arg183Gln, was first identified by paired whole-genome sequencing of affected and unaffected tissue. It was present in 88% (23/26) of patients with Sturge–Weber syndrome and in 92% (12/13) of those with an apparently non-syndromic port-wine stain, at a low VAF of 1.0–18.1% [11].

Independent sequencing recovered the same variant in 80% (12/15) of sporadic cases [18]. Since the mutant cells are heavily diluted by normal tissue, a negative result is usually an artifact of assay sensitivity rather than evidence against the diagnosis. Digital PCR, which counts single DNA molecules, recovered the variant below 1% in two of three previously negative patients [19]. Within *GNAQ*, it is the codon rather than the gene that defines the disease, because other codon 183 substitutions occur in capillary malformations lacking p.R183Q; the cross-gene extension of this argument rests on *GNA11*, discussed below.

Not every capillary malformation, however, is a Gq/11 lesion. In targeted sequencing of 82 lesions carrying neither a *GNAQ* nor a *GNA11* variant, pathogenic somatic variants in *PIK3R1* or *PIK3CA* were found in 9 of them. These nine lesions shared one appearance: a pale capillary malformation with visible dilated veins. Endothelial cells isolated from two *PIK3R1*-mutant lesions showed increased AKT phosphorylation, which decreased under PI3K, AKT or mTOR inhibition [20]. A single morphological label can therefore span two of the axes used in this review, and the pathway followed by a capillary malformation cannot be read from its name.

*GNA11* carries p.Arg183Cys in three of eight *GNAQ*-negative lesions with limb overgrowth [12,21]. It predominates in phakomatosis pigmentovascularis [22,23] and produces a variant Sturge–Weber phenotype [24]. Within the lesion, the variant is located in the endothelium. The allele frequency there reaches 3–43%, compared with 2–11% in bulk tissue. It is absent from the PDGFRB-positive cells of the perivascular coat [12,25], including the microdissected choroidal vessels of an enucleated eye [26].

### 4.2. A Weakly Activating Allele with Several Outputs

Codon 183 encodes the GTPase-regulatory residue, the amino acid that switches Gαq off once it has been activated. Substituting it therefore leads to persistent Gαq output, a property demonstrated in transfected cells long before any lesion was genotyped [27]. Endothelial cells expressing p.R183Q show constitutively active PLCβ3, the enzyme immediately downstream of Gαq, as well as activation of PKC, NF-κB and calcineurin, and increased ANGPT2. Angiopoietin-2 mediates part of the phenotype, and knocking it down normalizes the caliber of the vessels these cells form in mice [28].

A second arm runs to MAPK through RasGRP3 under PKC control [29], and a third runs to YAP, a transcriptional coactivator, through a Trio-Rho/Rac circuit independent of phospholipase Cβ [30,31]. These arms are separable: the barrier defect is rescued partly by ANGPT2 knockdown and, independently, by MEK1/2 inhibition [32]. Endothelial expression alone is sufficient to produce disease, with increased proliferation of the mutant endothelial cells in that model [33], and the result is progressive dilatation of immature venule-like vasculature, with aberrant MAPK and PI3K activation and no proliferative component [34].

### 4.3. Signal Strength: Why Codon 183 and Codon 209 Give Different Diseases

The two mutable codons differ in signal strength, and the diseases follow this difference. In a reporter assay comparing wild type, R183Q, Q209R, Q209L and a null allele, Q209L gave the strongest signal, while R183Q and Q209R were significantly weaker [35]. The weakness of the codon 183 allele is consistent with a separate assay of the port-wine-stain variant, in which mutant Gαq produced only a modest increase in extracellular signal-regulated kinase (ERK) activity [11]. Codon 209, by contrast, is the oncogenic residue of melanocytic neoplasia: it is mutated in 83% of blue nevi and 46% of uveal melanomas, exclusively at that position [36].

Uveal melanoma shows how consistently this codon is used. *GNA11* Q209 is found in 32% of primary uveal melanomas and *GNAQ* Q209 in 45%, whereas R183 alleles appear in only 6% [37]. The event occurs early [38] and is already present in benign precursors, with 15 of 16 choroidal nevi carrying a mutation in a subset of nevus cells [39]. R183 therefore dominates the malformations and Q209 the neoplasms [35].

In the choroid this distinction becomes clinically legible, because the two choroidal hemangiomas are separated by codon rather than by histology. The diffuse lesion carries *GNAQ* p.Arg183Cys, which was also found in three of three port-wine stains. The solitary, or circumscribed, lesion—the two names denote one entity—has been sequenced in two series that conflict at the allele level: one reported c.626A>T, p.Gln209Leu, in six of six cases [40], whereas the other found a different substitution at the same nucleotide, c.626A>G, predicted p.Gln209Arg, in 28 of 33 lesions (85%) sequenced at over 15,000-fold depth [41]. The allele distribution within codon 209 therefore remains unsettled; what is consistent across series is the codon itself.

The available molecular data therefore support a biological distinction between the two entities. Diffuse choroidal hemangioma is a congenital malformation associated with facial capillary malformation, whereas circumscribed choroidal hemangioma usually presents as an isolated lesion in adulthood. However, molecular evidence for the diffuse form remains limited [26].

The circumscribed lesion uses the same codon as uveal melanoma; where the allele is *GNAQ* Q209R, it is one that melanoma rarely uses, although the discordant Q209L report described above—the canonical melanoma allele—means that this contrast is allele-specific rather than absolute [37,41]. Codon identity alone therefore does not define malignant potential; the lineage and developmental context also matter, as shown by mouse models in which the same Q209L allele produced different melanocytic lesions depending on when and where it was expressed [42]. Codon 209 variants also recur under other names, including papillary hemangioma and extra-axial cavernous hemangioma [43,44].

### 4.4. Congenital Versus Infantile Hemangioma: The Suffix Is Uninformative

Despite its name, congenital hemangioma belongs to the Gq/11 axis. In the defining series, all eight lesions carried mutually exclusive mosaic variants affecting Gln209 in *GNAQ* or *GNA11*, with allele frequencies of 3–33%. The same driver was found in rapidly involuting and non-involuting lesions, so the genotype does not explain postnatal behavior; that behavior remains the basis of the RICH/NICH/PICH classification [10].

Infantile hemangioma shares the suffix but not the pathway, and its receptor-imbalance mechanism is presented in Section 9. Glucose transporter 1 (GLUT1) separates it from congenital hemangioma and from malformations: it was strongly positive in 139 of 143 infantile hemangiomas and absent from 66 vascular malformations [17,45,46]. Drug response also follows the pathway rather than the name. Propranolol was effective in 60% of infantile hemangiomas, compared with 4% on placebo [47], whereas the Gq-driven choroidal lesion of Sturge–Weber syndrome has responded poorly in a single enucleated globe, a finding its authors describe as preliminary [26]. The shared suffix therefore predicts neither the molecular driver nor the treatment response.

### 4.5. Ocular Sturge–Weber Disease and the Status of the Glaucoma Account

Glaucoma in Sturge–Weber syndrome has long been explained by elevated episcleral venous pressure. In a classic series, episcleral hemangiomas were visible in every glaucomatous eye, and episcleral venous pressure was elevated in every glaucomatous eye measured [48]. Molecular studies have now placed this hemodynamic mechanism within genetically abnormal tissue: abnormal scleral and episcleral tissue from Sturge–Weber eyes with glaucoma carries *GNAQ* p.R183Q, and the lesional vascular endothelium shows increased phospho-ERK and phospho-JNK compared with glaucoma controls [49].

The same mutant vessels also show abnormal arteriovenous specification through Notch signaling. They co-express arterial and venous markers instead of acquiring a stable identity, and episcleral vessel diameter correlates with the *GNAQ* variant allele frequency. This finding provides an early dose–response link between the mosaic burden and the vascular abnormality that can raise intraocular pressure [50].

The old and the new explanations are therefore complementary. Pressure rises through abnormal episcleral drainage, but the draining tissue itself is mosaic and molecularly active. Whether the conventional outflow pathway is also mutant remains unknown, because the variant has been demonstrated in the sclera, episclera and choroid, but not in the trabecular meshwork or Schlemm’s canal. Recent reviews therefore keep several mechanisms and add possible contributions from *GNA11*, GNB2 and macrophages [51].

### 4.6. Molecular Differences Between Diffuse and Circumscribed Choroidal Hemangioma

The molecular distinction between the two choroidal hemangiomas—codon 209 in the circumscribed lesion, codon 183 in the diffuse [26,40,41]—is presented in Section 4.3 and is decisive for classification. The two also behave differently from the moment they are detected. A systematic review of 106 studies and 3854 patients with circumscribed choroidal hemangioma found decreased vision in 90%, a subfoveal location in 48% and subretinal fluid in 84% [52], which means that the lesion is symptomatic through exudation rather than through mass effect. The diffuse lesion, in contrast, is often missed on routine examination and referred late [53].

At present, however, this molecular distinction has not influenced clinical management. Photodynamic therapy is currently the treatment of choice for the circumscribed lesion, with photocoagulation, thermotherapy, radiotherapy and anti-vascular endothelial growth factor (VEGF) injection as alternatives [54]. In a series of 172 patients managed at one center, observation was used in 30.2%, transpupillary thermotherapy in 52.9% and argon laser in 8.7%, with anatomical stability in 87.1% of observed eyes and improvement in 60.5% of treated eyes [55]. None of these approaches selects the driver. A lesion whose growth depends on constitutive Gq signaling is treated by destroying it or by draining its exudate, and no trial checked if inhibiting the pathway would work instead.

## 5. The PI3K/AKT/mTOR Axis and *TEK*/TIE2

### 5.1. Two Receptor-Proximal Genes, One Pathway, One Lesion Class

*TEK* and *PIK3CA* converge on PI3K-AKT-mTOR, and it is this convergence that makes the slow-flow lesions a single class. The first of them is *TEK*, the gene encoding the endothelial receptor TIE2, which was found through inherited venous malformation. A p.Arg849Trp substitution segregates with the disease, and twelve further families established the general property of these alleles: ligand-independent receptor hyperphosphorylation [56,57]. These variants result in ligand-independent receptor autophosphorylation and constitutive downstream signaling.

Somatic *TEK* variants are responsible for approximately half of venous malformations: they were present in 28 of 57 individuals (49.1%) in one series and in 17 of 30 (56.7%) resected unifocal lesions in another. The recurrent findings were a p.Leu914Phe substitution and double cis mutations, that is, two variants on the same copy of the gene; a somatic second hit abolishing TIE2 function was also found in an inherited lesion [58,59]. The architecture tracks the allele configuration: unifocal disease usually carries p.L914F, whereas multifocal forms are predominantly double cis mutations, and the pair p.Thr1105Asn-p.Thr1106Pro recurs in blue rubber bleb nevus syndrome, in which a somatic *TEK* mutation was found in 15 of 17 individuals [60].

The downstream effect is chronic AKT activation that no longer depends on ligand binding, and it affects the vessel wall rather than only the endothelial lining. TIE2-mutant endothelial cells produce less PDGFB, which explains the poor pericyte coverage around the enlarged veins that defines the histology [61]. A fibrin-gel model reproduces both the enlarged vascular lumens and the pericyte loss from the mutation alone, which shows that no additional insult is required [62].

Mutant TIE2 also does not signal in the same way as the wild-type receptor. A kinase inhibitor only weakly reduced AKT signaling driven by the mutant receptor, while rapamycin reduced it more effectively [63]. Ligand signaling still remains relevant, however: in *PIK3CA*-driven lesions, recruited smooth muscle supplies ANGPT1, which further stimulates TIE2 [64].

*PIK3CA* occupies the other half of the same class, and the two genes are largely mutually exclusive. Hotspot variants account for 54% (27 of 50) of venous malformations that lack a *TEK* mutation, and three of them, c.1624G>A, c.1633G>A and c.3140A>G, comprise more than 92% of *PIK3CA*-mutant individuals. A p110α-specific inhibitor restores abnormal phenotypes in both *PIK3CA*- and *TEK-mutant* endothelial cells [65,66], which is the pharmacological demonstration that a single pathway is at work.

On the lymphatic side, *PIK3CA* is found in nearly all cases. Mosaic variants, present in some cells of the body but absent from others, were detected in 16 of 17 patients with isolated lymphatic malformation [67] and in 108 of 143 resected lesions (75.5%) [68]. A smaller, mutually exclusive group is instead driven by *NRAS* [69].

Sampling of small periocular specimens is governed by cell type and by VAF. The mutation is confined to a cell type: it was present in four of six lymphatic-malformation-derived endothelial cell lines but not in fibroblasts from the same lesions [70]. Mosaicism is also low, with 91 of 142 pathogenic variants falling below 10% allele frequency [71], so a small or diluted specimen may read as normal.

Orbital lymphatic disease has direct molecular support: *PIK3CA* variants were reported in CVMS-associated orbital/periorbital lesions [72] and subsequently in seven orbital lymphatic malformations [73]. The most common orbital lesion, however, is neither a *TEK* nor a *PIK3CA* lesion; it is driven by *GJA4*, and Section 8.1 gives the detection frequencies and the specificity data [13,14].

### 5.2. Overgrowth, Borderline Tumors and Lymphatic Specification

The germline-mosaic end of this axis forms a spectrum. A consensus workshop unified the somatic *PIK3CA* conditions as the *PIK3CA*-related overgrowth spectrum (PROS), and CLOVES (congenital lipomatous overgrowth, vascular malformations, epidermal nevi, skeletal and spinal anomalies) is defined within it by the presence of a vascular malformation. Severity tracks the class of mutation and its distribution in tissue rather than the identity of the gene, and related overgrowth can arise from variants in AKT3 and PIK3R2 as well [74,75,76]. Periocular involvement enters by two routes: non-hotspot variants predominate in CLOVES and PROS, whereas hotspot variants predominate in common or combined lymphatic malformations [68]. Orbital lymphatic disease may therefore present in isolation or within an overgrowth phenotype.

Mosaic activating *PIK3CA* variants have been identified in orbital lymphatic and lymphaticovenous malformations associated with cerebrofacial vascular metameric syndrome (CVMS) [72]. The analytes in that report were an excised periorbital lymphatic malformation, a partially resected orbital vascular malformation and cell-free DNA from lymphatic cyst fluid at 4.3% VAF, so one of the three genotypes rests on a fluid analyte rather than on lesional tissue. The sampled phenotype was lymphaticovenous with ipsilateral cerebral venous anomalies, not the retinal arteriovenous lesion of the Wyburn-Mason phenotype, so the syndromic label is shared while the genotyped compartment is not. These findings provide direct molecular evidence for orbital disease but do not establish the genotype of retinal racemose malformation (historically termed retinal racemose hemangioma). Kaposiform hemangioendothelioma and the Kasabach-Merritt phenomenon sit at the boundary with the Gq axis, because their genotype crosses the line between the two axes: a somatic activating *GNA14* mutation was identified in tufted angioma tissue with Kasabach–Merritt phenomenon, in a single case [77]. *GNA14* also recurs in extra-axial cavernous hemangioma, where p.Gln205Leu upregulates MAPK, whereas a *GNAQ* codon-209 allele in the same series upregulates PI3K-AKT-mTOR instead [44].

A lymphangiogenic, mTOR-associated program separates these tumors from the malformations. S6K1 and phospho-S6K1 were expressed in all of 18 lymphatic anomalies, whereas activated mTOR was detected in half of the kaposiform cases and in none of the lymphatic malformations [78]. Serum angiopoietin-2 was roughly 14-fold above control in kaposiform hemangioendothelioma when the coagulopathy was present, and not when it was absent [79].

The effect of a drug aimed at this axis depends on which branch it blocks. In human vascular tumors, the demonstrated readouts are mTORC1 readouts—activation of p70 S6 kinase and S6—and rapamycin suppresses these signals [80]. It does not, however, block the whole pathway: AKT-Ser473, which is increased in mutant lymphatic endothelial cells, is controlled by mTORC2, upstream of mTORC1 [70]. mTOR inhibition therefore affects only part of the signaling produced by an activating *PIK3CA* variant.

Lymphatic specification follows a separate program led by PROX1. Loss of PROX1 stops lymphatic budding from embryonic veins while blood-vessel formation remains intact [81], and VEGF-C sustains this program. In *PIK3CA* H1047R disease, VEGF-C drives hypersprouting and progressive microcystic disease, and in the matching mouse model the lesions regressed only when VEGF-C blockade was combined with mTOR inhibition—neither treatment worked alone [82]. This explains why mTOR inhibition alone may give incomplete responses.

## 6. The RAS/MAPK Axis

### 6.1. Arteriovenous Malformation Is a Somatic MAPK Disease

Fast-flow lesions carry activating variants in the canonical RAS-RAF-MEK cascade, and which gene is hit tells us less than which pathway is. Somatic *MAP2K1* variants were detected in 7 of 10 extracranial arteriovenous malformation specimens [83], and mosaic *KRAS*, *NRAS*, *BRAF* and *MAP2K1* variants were found across a group of 160 children with sporadic vascular malformations of unexplained cause [84]. Detection reaches 78.3% and 86.0% in the two largest series, in which *KRAS* and *MAP2K1* are the most common drivers, and head and neck lesions show the same spectrum [85,86,87].

Within this single morphological category, the genotype predicts behavior in a way the category itself cannot. *KRAS* variants were associated with severe extended facial lesions and frequent post-surgical relapse, whereas *MAP2K1* variants were associated with lip-limited disease [85]; somatic RAS variants were associated with a more severe clinical stage (65.6% against 40.0%) [86]. The affected compartment is endothelial, which is why whole tissue understates the variant: one *HRAS*-mutant lesion showed 5% allele frequency in whole tissue against 31% in isolated endothelium [88]. Lesions bearing tumor names carry the same drivers, as *MAP2K1* and *KRAS* variants recur in intramuscular capillary-type hemangioma and epithelioid hemangioma [89,90].

The intracranial evidence is stronger. Somatic activating *KRAS* variants were found in 45 of 72 patients with sporadic brain arteriovenous malformation, but in none of 21 paired blood samples [91], which shows that the variant is confined to the lesion. Pooled data from 1726 patients put the frequency at 55% for *KRAS* and 7.5% for *BRAF* [92,93].

How far this generalizes is answered by sufficiency experiments, and the answer points to the pathway. Endothelial gain-of-function Kras, *BRAF* V600E, *MAP2K1* p.Lys57Asn and RIT1 variants are each sufficient to produce arteriovenous shunting in mice or zebrafish. In each model the lesions depend on MEK signaling regardless of the mutated gene: they resist PI3K inhibition and rapamycin while responding to MAP kinase inhibition [94,95,96,97,98,99,100].

The developmental logic is consistent with this. ERK activation pushes the endothelium toward an arterial identity and disrupts arteriovenous patterning through Delta/Notch and semaphorin signaling [101]. Active endothelial Notch4 can also produce shunts with arterialized veins, and these shunts regress when the transgene is switched off [102], so the shunt architecture is not fixed permanently.

### 6.2. The Germline Syndromes and Their Second Hits

Two capillary malformation-arteriovenous malformation (CM-AVM) syndromes place the same pathway under germline control. CM-AVM1 was defined by heterozygous inactivating *RASA1* mutations in six families with multiple small round-oval pinkish-red capillary malformations [103]. The gene encodes p120-RasGAP, the protein that restrains Ras signaling, and the genotype is specific to that phenotype: distinct mutations are numerous within the syndrome, 58 of them across 68 CM-AVM families, but none were found among 100 patients with common port-wine stain, 37 with Sturge–Weber syndrome or 24 with isolated arteriovenous malformation [104]. The spectrum extends to Parkes Weber syndrome and vein of Galen malformation [105,106].

CM-AVM2 is caused by germline loss-of-function *EPHB4* mutations, which were sought there because p120-RasGAP is a direct effector of *EPHB4*. This finding places both syndromes on one *EPHB4*-RAS-ERK axis and explains why *RASA1* accounts for only half of the phenotype [107], a phenotype that can mimic hereditary hemorrhagic telangiectasia [108].

Lesion formation requires a somatic second event in addition to the inherited one. An inactivating second hit, c.1534C>T, p.Arg512, was localized in endothelial cells of a capillary malformation [104,109] and lay in trans with the germline c.2125C>T, p.Arg709, that is, on the other copy of the gene. Biallelic somatic inactivation alone can also suffice [110], and mosaic *RASA1* mutations at 3–25% allele frequency explain part of the quarter of patients negative on germline testing [111].

The two syndromes converge mainly on Ras output. A catalytically inactive *RASA1* knockin reproduces the null phenotype, which shows that Ras-GAP activity is the key function in this setting [112]. Loss of *RASA1* also impairs collagen IV export and promotes endothelial death [113]. In lymphatic endothelial cells, the EPHB4-*RASA1* complex limits the Ras-MAPK activation triggered by the PIEZO1 shear-stress sensor, and this restraint is required for lymphatic valve specification [114,115]; *RASA1* also suppresses endothelial mTORC1 [116].

Ephrin-B2 and EphB4 also help to separate arterial from venous endothelium under flow. In human arteriovenous malformation tissue, ephrin-B2 marks the arterial side and EphB4 the venous side [117,118]. A zebrafish model adds an important qualification: in rasa1 mutants, the shunts begin on the venous side [119]. CM-AVM should therefore be understood as a Ras-MAPK disease of endothelial flow sensing and arteriovenous identity, rather than simply as an arterial lesion.

### 6.3. Pyogenic Granuloma: A Reactive Lesion with a Clonal Driver

Pyogenic granuloma, or lobular capillary hemangioma, behaves as a reactive, trauma-associated and often self-limited proliferation, yet it carries oncogenic drivers. Here the clinical category and the molecular cause come apart. *BRAF* p.Val600Glu is the major driver of the form that arises secondarily on a port-wine stain: it was present in 8 of 10 such lesions and was localized to endothelial cells by mutation-specific immunohistochemistry [120]. These lesions retain the underlying *GNAQ* c.548G>A, so they arise from cells of the stain and carry a second MAPK hit on a Gq-mutant field [120].

Sporadic lesions are different: the same study found *BRAF* c.1799T>A in only 3 of 25 [120], and childhood lesions carry activating MAPK variants in 4 of 15 (27%) [121]. The genotype is site-dependent, and the periocular lesion stands apart molecularly. Pyrosequencing of 28 conjunctival, eyelid and orbital specimens found no *BRAF* V600E, although eyelid nevi analyzed in parallel were mutant in 3 of 3 [122,123], which shows that the method was able to detect the variant when it was present.

The RAS genes and *MAP2K1* have never been sequenced in periocular lesions, so that negative dataset has no positive counterpart. Pathway activation alone may be sufficient, since the lesion has arisen de novo under selective BRAF-inhibitor therapy in three reported patients, one of whom was also taking a retinoid, and the authors call for confirmation [124]. In one of its forms, then, a lesion whose clinical identity rests on reactivity is in fact a clonal endothelial neoplasm with a canonical oncogene; elsewhere the same name may cover no identifiable driver at all.

## 7. Convergence of the Three Cytoplasmic Cascades

The three-axis division is useful, but the axes are not independent, and at their points of overlap the same phenotype can arise from different genotypes. Mutant Gαq reaches ERK through PKC and RasGRP3 [29], so a Gq lesion also has a MAPK component. The barrier defect in *GNAQ* p.R183Q endothelium improves additively with MEK inhibition and ANGPT2 knockdown [32], which means that neither pathway explains the leak on its own. Port-wine stain endothelium likewise shows combined MAPK and PI3K activation [34].

Signaling also travels in the opposite direction: one Gq/11 codon-209 allele upregulates PI3K-AKT-mTOR, whereas a *GNA14* allele in the same series upregulates MAPK instead [44], so the gene alone does not predict which cascade dominates. *RASA1* suppresses endothelial mTORC1 downstream of *EPHB4* [116], and losing it therefore releases growth signaling as well as Ras-MAPK. *NRAS* Q61R in kaposiform lymphangiomatosis over-activates both cascades at once [125].

**Figure 2:**
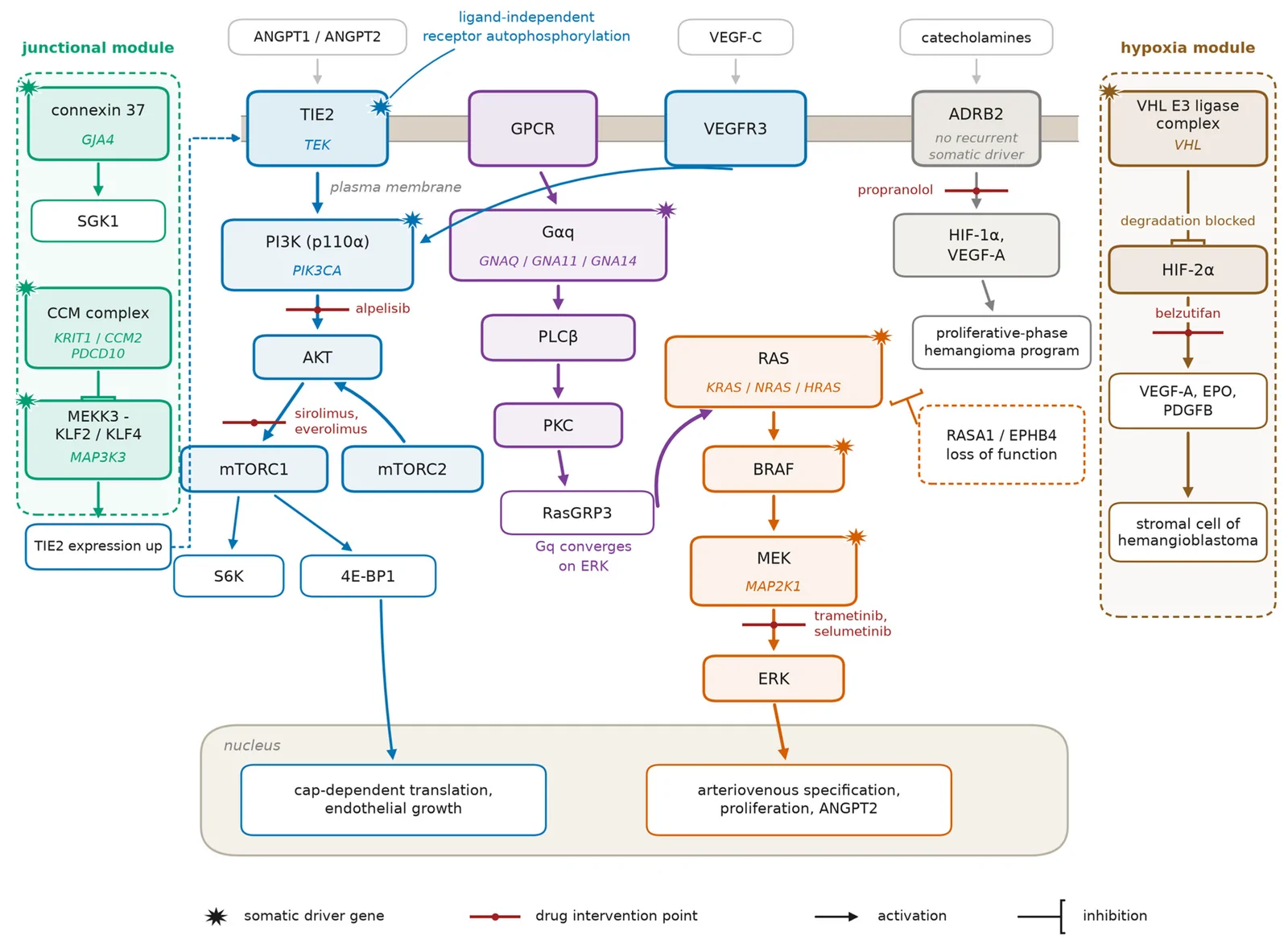
The signaling axes that drive periocular and orbital vascular anomalies converge on a small number of nodes, and every current targeted drug acts at one of them. Receptor-to-nucleus schematic of the four connected axes and two separable modules, drawn with the pathway colors of Figure 1. Starburst glyphs mark nodes at which somatic driver variants have been reported in vascular anomalies, labeled with the gene symbol in italics; red bars mark drug intervention points. TIE2, encoded by *TEK*, is shown with ligand-independent autophosphorylation, the mechanism by which recurrent somatic *TEK* variants such as p.Leu914Phe activate the receptor in venous malformation [58,59,61]. PI3Kα, encoded by *PIK3CA*, signals through AKT to mTORC1, whose substrates S6K and 4E-BP1 are drawn below it; mTORC2 is placed upstream of AKT, which it phosphorylates at Ser473. S6K activation has been demonstrated in lymphatic anomalies and vascular tumors by immunohistochemistry [78,80]. Gαq, encoded by *GNAQ*, *GNA11* or *GNA14*, activates PLCβ and PKC, and PKC signals through RasGRP3 to RAS, which is how the Gq axis converges on ERK [28,29]. The RAS-BRAF-MEK-ERK cascade is drawn with its physiological brake, the EPHB4-RASA1 complex, whose loss of function releases Ras-MAPK activation [103,107,117]. The junctional module shows the connexin 37 hemichannel encoded by *GJA4* signaling through SGK1 [13,126], the CCM complex whose loss releases MEKK3-KLF2/KLF4 signaling [127,128,129,130,131], and the induction of TIE2 expression that links the two [132]. The hypoxia module shows loss of the VHL E3 ligase complex, stabilization of HIF-2α and the resulting transcriptional output in the stromal cell of hemangioblastoma [133,134,135]. The beta-adrenergic arm carries no starburst because propranolol acts on infantile hemangioma, in which no recurrent somatic driver has been identified; its effect has been linked to collapse of the HIF-1α and VEGF-A program [136]. Drug intervention points shown are alpelisib at PI3Kα, sirolimus and everolimus at mTORC1, trametinib and selumetinib at MEK, and propranolol on the beta-adrenergic arm. Belzutifan is drawn below HIF-2α, on the arrow to its transcriptional targets, because it antagonizes HIF-2α itself and does not restore the VHL-mediated degradation that the germline variant has abolished. Abbreviations: ANGPT, angiopoietin; VEGF, vascular endothelial growth factor; VEGFR3, vascular endothelial growth factor receptor 3; ADRB2, β2 adrenoceptor; GPCR, G-protein-coupled receptor; PLCβ, phospholipase C beta; PKC, protein kinase C; mTORC1 and mTORC2, mechanistic target of rapamycin complexes 1 and 2; S6K, p70 S6 kinase; 4E-BP1, eukaryotic translation initiation factor 4E-binding protein 1; SGK1, serum/glucocorticoid-regulated kinase 1; MEKK3, mitogen-activated protein kinase kinase kinase 3; KLF, Kruppel-like factor; CCM, cerebral cavernous malformation; VHL, von Hippel–Lindau; HIF, hypoxia-inducible factor.

From the therapeutic side, the crosstalk is asymmetric, because inhibiting one arm does not substitute for inhibiting the other. Kras-driven arteriovenous malformation depends on MEK and resists PI3K inhibition [94], and *NRAS*-driven endothelial change is prevented by MAP kinase inhibition but not by rapamycin [100]. The overlap is explained downstream: ERK output, mTORC1 activity, angiopoietin-2 and Notch-dependent arteriovenous specification are nodes shared by all these drivers. Consequently, similar vascular phenotypes may arise from alterations in different signaling pathways, limiting the ability of morphology alone to predict the underlying genotype. Figure 2 draws these axes as one signaling map, with the nodes at which somatic drivers arise and the points at which the drugs act.

## 8. Endothelial Junctional and Mural Signaling

### 8.1. Connexin 37 and the Orbital Cavernous Venous Malformation

The traditional term ‘cavernous hemangioma’ is increasingly difficult to reconcile with the molecular and histological features of this lesion. The lesion carries one recurrent somatic driver, *GJA4* c.121G>T, p.Gly41Cys, which deep sequencing found in 25 of 26 lesions (96.2%) in the discovery cohort, at a higher VAF in sorted endothelial fractions than in whole tissue [13]. This gradient places the mutation in the endothelial cell itself.

An independent series confirmed the association and confined it: *GJA4* mutation was present in 44 of 59 genotyped orbital cavernous venous malformations and absent from all orbital varices, infiltrating venous malformations and arteriovenous malformations tested [14]. The lower yield relative to the discovery cohort may reflect differences in sequencing depth and endothelial enrichment, as well as case definition, since the discovery cohort was selected histologically whereas the confirmatory series was clinicopathological [13,14]. Among orbital vascular malformations, the genotype therefore tracks the cavernous phenotype specifically, including the lesion the orbital surgeon meets most often—in that series of 92 orbital lesions, 90 were vascular malformations and 60 of the 90 had the cavernous phenotype [14].

The same allele crosses organs and morphological labels. It occurs in 12 of 14 hepatic lesions called hemangiomas and in three of five cutaneous venous malformations, in which mutant connexin 37 activates SGK1 non-canonically and spironolactone reverses the change [126]; it also drives intracranial extra-axial cavernous hemangioma with the same SGK1 dependence [137]. Lesions named differently in different organs may therefore share one mechanism.

The allele also appears in the orbit outside the cavernous venous malformation. An orbital angioleiomyoma carried the same *GJA4* p.Gly41Cys variant, and on magnetic resonance imaging it resembled a cavernous venous malformation closely enough to be reported as one; only immunohistochemistry together with molecular testing established the diagnosis, although it remains casuistic [138]. The genotype narrows the orbital differential without closing it, and histology is still needed alongside the sequence.

The mechanism fits a dilated, non-proliferative vessel. The mutant forms a hyperactive hemichannel on voltage clamp and weakens endothelial integrity when overexpressed, and carbenoxolone reverses that effect [13]. Normal connexin 37 helps to keep the endothelium quiescent and arterial, downstream of shear-activated NOTCH [139]. When this stabilizing signal is lost, the vessel can enlarge without becoming a proliferative tumor: connexin 37 is absent where vessels expand, and Cx37/Cx40 double-null mice develop wide congested channels resembling cavernous hemangioma [140,141]. The histology agrees: these lesions are GLUT1 negative, with multilayered desmin-negative mural cells and a Ki-67 index near zero, whereas orbital infantile hemangiomas are GLUT1 positive, with a single pericyte layer and a mean Ki-67 of 16.3% [46]. Orbital tissue has not been used to test whether the mutant drives endothelial aging or blocks quiescence, because mural coverage has not been measured against mutation status.

A lesion with no endothelial proliferation, an abnormal mural coat and a gain-of-function variant in a junctional gene is a malformation, not a tumor, and the correct name is orbital cavernous venous malformation; the oculoplastic terminology has been called inaccurate or controversial [142]. The name changes what the clinician expects: a report saying hemangioma implies a proliferating tumor for which antiproliferative therapy is rational, whereas a cavernous venous malformation is a static slow-flow network whose enlargement is hemodynamic rather than mitotic.

The treatment implication is less clear. Spironolactone and SGK1 inhibition are so far supported only by cell and mouse data, and because these lesions barely proliferate, it is uncertain whether a drug aimed at growth would have a meaningful target at all.

### 8.2. The CCM Complex and Its Uncertain Ocular Counterpart

Cerebral cavernous malformation is the second junctional paradigm, and a warning about its ocular counterpart. *KRIT1*/CCM1, *CCM2* and *PDCD10*/CCM3 are loss-of-function genes, and a lesion arises only when one of them is lost completely, through a Knudsonian two-hit mechanism in which both copies of the gene are inactivated. The somatic hit is confined to a subset of the endothelial cells lining the cavernous vessels [127,128,129,130].

The affected endothelium then gains MEKK3-KLF2/4 signaling, a kinase-to-transcription-factor program that the intact CCM complex normally holds in check. Deletion of Map3k3, Klf2 or Klf4 prevents lesions in mice, which places this program in the causal path [131].

Sporadic disease is instead largely explained by somatic activators, and there the genotype maps onto the imaging phenotype. *MAP3K3* p.Ile441Met occurred in 22 of 23 popcorn-like lesions, but in only 1 of 40 subacute-bleeding or multifocal lesions [143], so the imaging appearance carries genotypic information. Growth appears to require a third hit: gain-of-function *PIK3CA* accompanies biallelic CCM loss in the same endothelial cells in 75% of sporadic lesions that already carry a *KRIT1*/*CCM2* or *MAP3K3* mutation [144,145].

The ophthalmic counterpart is much less certain. Retinal cavernous hemangioma is usually treated as an ocular sign of familial CCM disease, so it often leads to intracranial imaging. The diagnosis, however, still rests on the fundus appearance and family history rather than on genotyping [146]. No study has sequenced retinal lesional tissue for *KRIT1*, *CCM2*, *PDCD10*, *MAP3K3* or *PIK3CA*. It is therefore unknown whether the retinal lesion follows the two-hit CCM mechanism, the three-hit mechanism described in some intracranial lesions, or a different route altogether. The genotype-imaging correlation established in the brain has not yet been tested in the eye.

## 9. Hypoxia Signaling and the Retinal Hemangioblastoma

### 9.1. VHL, HIF-2α and the Retinal Lesion

The *VHL*-driven lesions of the retina activate a transcriptional program without the stimulus that normally triggers it. The mechanism is pseudohypoxia: the cell behaves as if it lacked oxygen, although the oxygen supply is normal. Both HIF-1α and HIF-2α proteins are constitutively stabilized under normoxia in *VHL*-deficient cells, and re-introduction of functional *VHL* makes them unstable again, which identifies the loss of *VHL* protein as the cause [133,134].

It is the stromal cell of the hemangioblastoma that produces this output, since VEGF mRNA overexpression in that cell correlates closely with elevated HIF-2α [134,135]. The neoplastic cell itself appears to be an embryologically arrested mesodermal hemangioblast, because it co-expresses brachyury, Flk-1 and Scl [147]. This evidence, however, comes from 31 central nervous system hemangioblastomas with no retinal lesion among them, so extending it to the retinal lesion is an inference rather than a finding. The vascularity of the lesion follows from the transcriptional effect of the lost allele, not from a real shortage of oxygen.

Genotype stratifies the ocular risk within this single gene. Retinal involvement is common but not universal—it was present in 335 of 890 patients in the NIH cohort. Complete deletion of *VHL* carried the lowest prevalence, 14.5%, and the best mean visual acuity [148], whereas truncating variants carry a heavier burden (0.10 versus 0.05 lesions per year of life) and a shorter median enucleation or phthisis-free survival (56 versus 78 years) [149].

The position within the protein matters more than the class of mutation. Missense variants at the HIF-1α binding site behave like truncating variants and produce more affected eyes and more large lesions than missense variants elsewhere in pVHL [150]. Two patients with the same diagnosis but different variants therefore do not carry the same ocular risk.

A worldwide cohort of 1350 carriers across 40 centers has since confirmed that the risk is mutation-specific rather than class-specific. Among the six most common variants, 47 of 90 pairwise comparisons differed significantly, and every comparison between different codons differed except one pairing a truncating with a missense variant [151]. Ocular risk therefore appears to be associated more closely with the affected codon than with broad mutation class. The consequence concerns surveillance and not only prognosis, because the interval at which an eye is examined can be set according to the variant the patient carries [152,153].

Belzutifan inhibits the terminal effector of this axis, and its trial confirms the role of the axis in the eye while showing the cost of pathway-directed therapy. It improved all 16 evaluable eyes of the 12 patients with retinal hemangioblastoma in the phase 2 LITESPARK-004 trial, and responses also occurred in 15 of 50 patients with central nervous system hemangioblastoma (30%). The adverse effects were systemic, with anemia in 90% and fatigue in 66% [154]. The ocular improvement persisted over a median of 37.3 months with no new lesions [155].

This is the best ocular result for any targeted agent discussed in this review, but it comes from a single-arm subgroup of 16 eyes without a randomized comparator, and it is not known whether HIF-2α inhibition prevents new lesions or only shrinks those already present.

### 9.2. Infantile Hemangioma: A Hypoxic Program Without a Driver

Infantile hemangioma differs from the lesions discussed above because its cellular phenotype and molecular markers are well characterized, despite the absence of a recurrent somatic driver. GLUT1 immunoreactivity separates it from the malformations, based on the series quantified in Section 4.4 [45], and it forms part of a placental microvascular signature that also includes FcγRII, Lewis Y antigen and merosin [17]. The resemblance to placenta, however, is not a matter of origin, since genotyping of lesional endothelium matched the child and never the mother [156].

The origin of the lesion remains contested between two marker-based hypotheses from the same group: one derives it from a hemangioblast-derived hemogenic endothelium, the other from a placental chorionic villous mesenchymal core [157,158]. What is not contested is the behavior of the cell that starts the lesion. A CD133-selected hemangioma stem cell reproduces the whole life cycle: it generates GLUT1 positive human vessels in immunodeficient mice, and these are replaced by adipocytes by 2 months [16].

The signaling defect is an imbalance of receptors rather than an excess of ligand. VEGFR1, encoded by *FLT1*, is markedly reduced through loss of NFAT-dependent transcription, which leaves VEGFR2 signaling constitutively active. Germline variants in *KDR* and in *ANTXR1*, encoding TEM8, are present in a subset of patients [15]. The hypoxia program is also active, with HIF-1α, VEGF-A, MMP-9 and SDF-1α elevated during the proliferative phase [159].

Despite all this, no somatic driver mutation has been identified in infantile hemangioma, and for this reason the lesion cannot be placed on the same genotype-first axis as the *GNAQ*, *GJA4*, *TEK* and *PIK3CA* lesions.

Propranolol came from a clinical observation rather than from this biology, and the two have still not been reconciled. It entered practice by chance and was validated only afterwards: a randomized double-blind trial of 3 mg/kg/day for 6 months gave complete or near-complete resolution in 60% versus 4% on placebo [47,160]. The efficacy is therefore well established, but the mechanism is not.

## 10. Genotype–Phenotype Correlation

The driver gene alone does not specify the lesion; if it did, the classification would only replace morphological names with gene names. The effect of a somatic variant depends instead on three factors: when in development it arose, in which lineage it arose, and how strongly the mutant allele signals.

**Figure 3:**
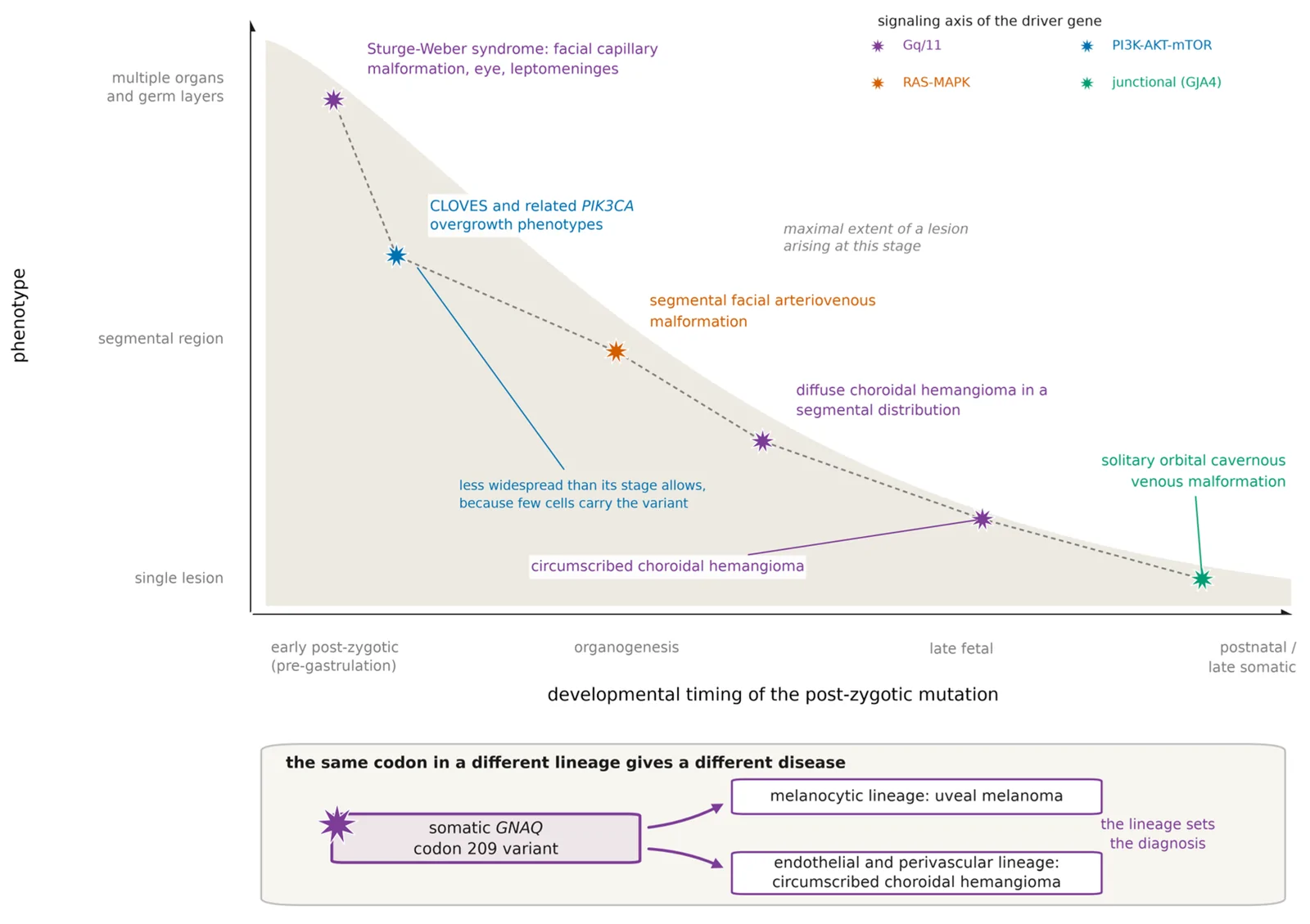
The developmental timing of a post-zygotic mutation, not the identity of the mutated gene, sets how much of the body the resulting phenotype occupies, and the lineage in which the mutation lands sets which disease it becomes. Schematic; the axes are ordinal and deliberately carry no numeric scale, and the shaded wedge indicates the phenotype breadth attainable by an event arising at each stage rather than a measured quantity. Starbursts mark example lesions, colored by pathway axis as in Figure 1 and Figure 2, positioned according to the developmental window in which the causative event is inferred to occur: earliest events give multi-organ segmental disease spanning skin, eye and leptomeninges in Sturge–Weber syndrome and multi-tissue overgrowth in the *PIK3CA*-related spectrum including CLOVES [11,75,164], intermediate events give a segmental regional lesion, and the latest events give an isolated single lesion such as a circumscribed choroidal hemangioma or a solitary orbital cavernous venous malformation [13,41]. The ordering shown is supported experimentally. In mice, ubiquitous blastocyst-stage expression of *GNAQ* p.Arg183Gln caused 100% embryonic death, with vascular defects in most of those embryos, whereas mosaic expression allowed survival without obvious vascular defects, so the phenotype depends on a narrow window of expression [7], and identical *GNAQ* p.Gln209Leu induced at successive embryonic days or in different lineage-restricted Cre lines produces different lesions [42]. The lower panel shows the lineage dimension for a single codon: somatic *GNAQ* codon 209 variants in a melanocytic lineage give uveal melanoma [36,37,38], whereas in an endothelial or perivascular lineage the same codon gives circumscribed choroidal hemangioma, at an allele that uveal melanoma almost never uses (an allele-specific contrast: it holds for p.Gln209Arg, not for the canonical melanoma allele p.Gln209Leu, also reported in this lesion; see Section 4.3) [40,41]. Abbreviations: CLOVES, congenital lipomatous overgrowth, vascular malformations, epidermal nevi and skeletal anomalies.

Timing determines the extent of disease. Mice expressing *GNAQ* p.Arg183Gln in every cell from the blastocyst stage all died as embryos, with vascular defects matching the human phenotype, whereas mosaic expression allowed some mice to survive. This formally validates Happle’s paradominant model for Sturge–Weber syndrome, which holds that such a lesion can exist only in mosaic form [7]. An event arising before the skin, leptomeninges, choroid and sclera separate will therefore affect all of them, while a later event gives an isolated lesion. Figure 3 plots this relation, with example lesions placed by the developmental window in which the variant is presumed to arise.

The human data agree. With an ultra-sensitive assay, *GNAQ* c.548G>A was found in blood or saliva DNA in 4 of 40 patients with Sturge–Weber syndrome [19], which means that in a minority of patients the mutation arose early enough to reach the hematopoietic lineages. Timing also determines which lesion develops: the identical *GNAQ* Q209L allele produced leptomeningeal melanocytomas, spinal melanoma or dermal blue-nevus-like lesions, depending on the embryonic day at which it was induced and the lineage in which it was switched on [42].

Lineage is the second factor, and the ocular tissues show it clearly, because one codon gives different diseases in different lineages. Codon 209 substitutions drive melanocytic neoplasia at the prevalences given in Section 4.3 [37], and they act early, being present in nearly all choroidal nevi but only in a subset of the nevus cells, as quantified in Section 4.3 [39].

The same codon in a vascular lineage gives a different lesion. Most circumscribed choroidal hemangiomas in the larger series carried *GNAQ* c.626A>G, predicted to give p.Gln209Arg, an allele that uveal melanoma almost never uses, although a smaller series reported p.Gln209Leu—the canonical melanoma allele—in six of six cases, so the allele distribution within codon 209 remains unsettled; codon identity alone therefore does not define malignant potential. In capillary malformation the same gene acts in the endothelium: the mutation was confined to the endothelial fraction of sorted lesions and absent from the PDGFRB-positive cells surrounding the vessels [12].

Signal strength separates malformation from tumor within one gene, and Section 4.3 gives the reporter-assay and choroidal-codon data. Codon 183 alleles activate the pathway weakly and dominate the malformations, whereas the strongly activating codon 209 alleles dominate melanocytic neoplasia [35,37,40]. The rule is not absolute, since *GNAQ* p.Gln209Arg was found in one of nine sequenced capillary malformations [35].

Allele strength governs how hard the pathway is driven per cell; how many cells carry the variant is a second dimension of the same variable, and within the eye this burden does appear to scale with severity, since episcleral vessel diameter correlates positively with variant allele frequency [50].

Orbital involvement is a separate question, and the orbit appears to have a genotype of its own. *GJA4* is confined there to the cavernous phenotype [14], and no somatic genotype has been reported for a periocular or orbital arteriovenous malformation. The intraocular lesions have different molecular associations: the choroidal hemangiomas are *GNAQ*-associated, whereas retinal capillary hemangioblastoma follows the VHL/HIF axis. The genotype of retinal racemose malformation is not established by the cited studies; the three *PIK3CA*-positive individuals in [72] had orbital lymphatic or lymphaticovenous malformations in CVMS. Absence of lesional sequencing should not be interpreted as a negative genetic result.

Timing matters clinically because the eye is often where an early event first becomes visible, and a periocular genotype therefore predicts what else to look for. Somatic *GNAQ* c.548G>A is detectable in affected tissue in the large majority of patients with Sturge–Weber syndrome and of those with an apparently non-syndromic port-wine stain, at the rates given in Section 4.1 [11]. A cutaneous lesion may therefore be isolated, or it may be the visible sign of leptomeningeal and ocular disease.

The gene involved points toward particular syndromes. *GNA11* mutation gives a phenotype of disseminated reticulate capillary malformation, limb hyper- or hypotrophy and glaucoma in two of three patients [24], and the same mosaic alleles underlie phakomatosis pigmentovascularis [22,23]. For *PIK3CA*, a consensus workshop grouped CLOVES syndrome and the other overgrowth entities into the *PIK3CA*-related overgrowth spectrum on the basis of genotype rather than morphology [75], and *PIK3CA*-positive orbital lymphatic and lymphaticovenous malformations have been reported in CVMS [72].

A capillary malformation with a CM-AVM distribution carries a different implication. *RASA1* mutations have been found in 68 CM-AVM families, but in none of 100 patients with common port-wine stain and none of 37 with Sturge–Weber syndrome [104], and germline loss of *EPHB4* explains much of the remainder [107]. The family to which a periocular lesion belongs therefore changes the systemic work-up, not only the local plan.

Some observations do not fit this reasoning. Why lesions carrying the same driver behave differently after birth is unknown: the same *GNAQ* or *GNA11* codon 209 variant occurs in both rapidly involuting and non-involuting congenital hemangioma, so the behavior that defines this nomenclature is not determined by the genotype [10]. Why the same *PIK3CA* hotspot produces a purely venous lesion in one patient and a lymphatic one in another is likewise unexplained. No study relates variant allele frequency to the magnitude or durability of drug response, so variant allele frequency has no established prognostic meaning.

Molecular characterization remains incomplete for several periocular entities, and established pathways should not be assigned to them without lesion-specific evidence. Orbital varix has no identified driver: the only orbital series to genotype varices found *GJA4* wild type in all eight [14], and no deep or single-cell sequencing has been applied to varix tissue. It may be a distinct genotype, a hemodynamic phenotype of normal vessels, or a lesion whose variant lies below the detection threshold. This remains undetermined, and the published literature concerns management rather than genotype [161].

The lymphatic-venous lesion in the 92-lesion orbital series was tested only for *GJA4*, *BRAF* and *KRAS* [14]. However, *PIK3CA*-positive orbital lymphatic and lymphaticovenous lesions have been reported in CVMS [72,73]; their frequency across unselected orbital subtypes remains uncertain. Conjunctival vascular and lymphatic malformation has no primary genotyping literature, and the two conjunctival datasets that exist address other lesions [122,162]. What does exist is natural history—two conjunctival lesions followed for decades without treatment, neither of which progressed [163].

**Figure 4:**
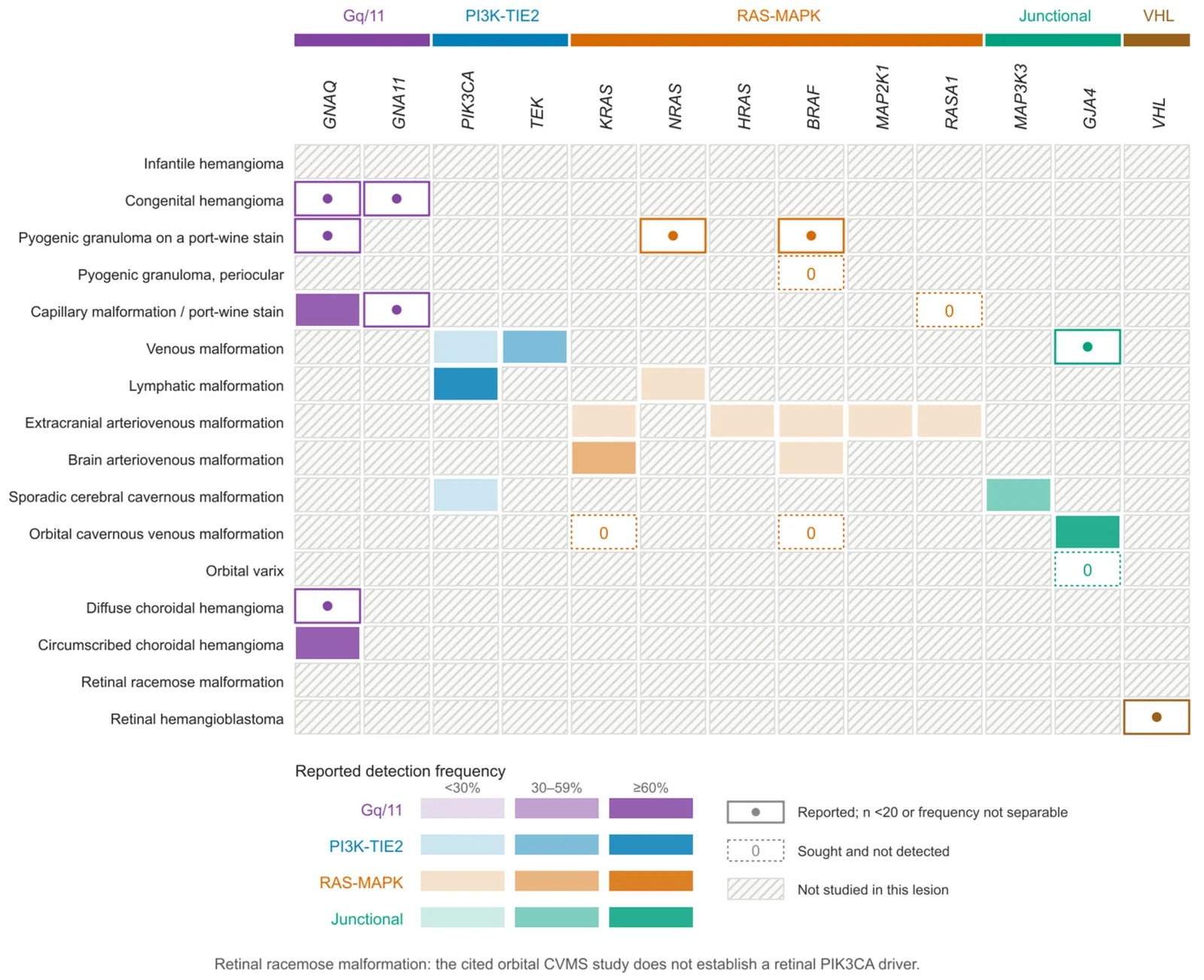
Reported driver genes cross lesion categories, and the hatched cells make visible how much of the gene-by-lesion space has not been examined at all. Matrix of driver gene (columns, grouped by pathway axis with the colors of Figure 1, Figure 2, Figure 3 and Figure 5) against lesion (rows). Cell shading gives the reported detection frequency band for that gene in that lesion, and the legend shows the three bands separately in each pathway color, since a band is read from the saturation of that color rather than from a single gray scale; the *VHL* axis carries no shaded cell because its one reported pairing has no separable frequency; open cells with a dot indicate that the gene has been reported in that lesion but in a cohort of fewer than twenty specimens or without a separable frequency; dashed cells containing zero indicate that the gene was specifically sought and not detected; hatched cells indicate that the pairing has not been studied, and are the majority of the matrix. Every filled cell rests on a specific report: *GNAQ* and *GNA11* codon 209 in congenital hemangioma [10]; *GNAQ* codon 183 in capillary malformation and port-wine stain, with *GNA11* in a minority [11,21] and *RASA1* specifically absent in common port-wine stain and Sturge–Weber syndrome [104]; *BRAF* p.Val600Glu with a single *NRAS* variant in pyogenic granuloma arising on a port-wine stain, in which the underlying *GNAQ* variant is retained [120], contrasted with the absence of *BRAF* p.Val600Glu in periocular pyogenic granuloma [122]; *TEK* and *PIK3CA* in venous malformation [66], with the same *GJA4* p.Gly41Cys allele reported in cutaneous venous malformations and hepatic lesions of identical histology [126]; *PIK3CA* and *NRAS* in lymphatic malformation [68,69]; *KRAS*, *MAP2K1*, *HRAS* and *BRAF* in extracranial arteriovenous malformation [86] with *RASA1* [85]; *KRAS* and *BRAF* in brain arteriovenous malformation [92]; *MAP3K3* and *PIK3CA* in sporadic cerebral cavernous malformation [143]; *GJA4* in orbital cavernous venous malformation [13], where *BRAF* and *KRAS* were sought and not found and where *GJA4* was absent from orbital varices [14]; *GNAQ* codon 183 in diffuse and codon 209 in circumscribed choroidal hemangioma [40,41]; and *VHL* in retinal hemangioblastoma [148,149]. Cells with a dashed border and a zero are reserved for pairings in which a study assayed that gene in that lesion; the two Gq/11 cells for infantile hemangioma are hatched, because the receptor-imbalance and placental-signature accounts cited for that lesion [15,16] did not sequence *GNAQ* or *GNA11*. Frequency bands are read from the cited cohorts; most rest on single cohorts rather than on meta-analysis, the exception being the brain arteriovenous malformation cell, which is pooled [92], and the matrix should be read as a map of what has been reported rather than as a set of population frequencies. The columns cover the genes discussed as pathway drivers here; *PIK3R1*, reported in capillary malformation with dilated veins [20], is not among them. The *PIK3CA* cell for retinal racemose malformation is hatched because the cited CVMS study genotyped orbital lymphatic or lymphaticovenous tissue, not retinal arteriovenous malformation [72].

No direct genotyping of retinal racemose malformation was identified in the cited studies; the orbital findings in CVMS [72] do not establish its molecular driver. Each of these lesions can be photographed directly by an ophthalmologist, so the absence of sequencing is a matter of tissue access and priority, not of biological inaccessibility. Figure 4 shows the same evidence as a matrix of driver gene against lesion, in which the hatched cells mark the combinations that have not been studied.

## 11. Molecular Diagnostics in Practice

The choice of material comes before any sequencing chemistry, and it affects every step that follows. These lesions arise from post-zygotic variants, that is, mutations acquired after fertilization and confined to a subset of lesional cells, so peripheral blood is the wrong primary sample.

In the largest assessment of testing for disorders of somatic mosaicism, the yield depended on three things: sequencing at approximately 2000× coverage, a broad gene set, and DNA from affected tissue. Blood served only as a comparator proving that a variant was somatic rather than inherited. Fresh and formalin-fixed paraffin-embedded tissue performed equivalently, whereas fibroblast culture enriched growth-advantaged variants such as *PIK3CA* [165]. Tissue in either form is therefore adequate, and the most common reason a diagnosis is missed is that no tissue was banked at orbitotomy.

The mutant cells make up only a small fraction of the lesion, so bulk sequencing of a small orbital biopsy averages the signal across tissue in which only the endothelium is informative. *PIK3CA* variants were confirmed in four of six endothelial cell lines derived from lymphatic malformation, while fibroblasts from the same lesions carried none [70]. In one *HRAS*-mutant arteriovenous malformation, the allele frequency was 5% in whole tissue but 31% in isolated endothelial cells [88]—a difference produced by the cell mixture alone.

Ophthalmic lesions show the same pattern. *GJA4* c.121G>T, p.Gly41Cys was enriched in the endothelial fractions of orbital cavernous venous malformation [13], and microdissected choroidal vessels from an enucleated Sturge–Weber globe carried *GNAQ* c.548G>A, p.Arg183Gln at 21.1% mutant allele frequency, against 0.5% in retinoblastoma choroid [26].

This dilution explains why standard clinical sequencing fails, because it is built around clonal tumor variants at 10% or above, and mosaic variants commonly fall below that threshold: 91 of 142 pathogenic mosaic variants across 319 vascular malformation samples lay below 10% variant allele frequency [71]. Deeper sequencing recovers them—a 129-gene panel at approximately 1% sensitivity gave an 80.8% diagnostic rate, with only 60% of variants reaching 5% [166].

Below 1%, depth alone stops working and enrichment chemistry is required. Peptide nucleic acid clamping blocks amplification of the normal allele so that the mutant one dominates the reaction, and combined with droplet digital PCR it recovered *GNAQ* c.548G>A in two of three patients previously scored negative by next-generation sequencing [19]. A broader panel, however, is less sensitive, and the two cannot be compared on sensitivity: a broad panel is scored by yield across many genes, 62.4% of 1197 patients, whereas digital PCR is scored against pre-specified hotspots [167,168]. The reported negative-panel proportion of 27.8% [169] and the approximately 42% of cases unresolved in a cohort with a diagnostic yield of 58% [165] describe the proportion of patients without a molecular diagnosis. They do not estimate assay sensitivity or false-negative rates, which require an independent reference standard.

Where tissue is unobtainable, the type of lesion determines which fluid to test. Plasma cell-free DNA, the fragmented DNA circulating free in blood, was informative in two of eight arteriovenous and one of three venous malformation patients [168]. It is therefore worth sampling in a fast-flow or venous lesion, although the allele frequency in draining-vein samples falls with distance from the nidus, the core of the lesion [170,171].

Lymphatic malformation behaves in the opposite way: cyst fluid was positive in seven of seven, whereas plasma was negative in all 26, including four of five never operated [168]. Lymphatic fluid is therefore the analyte to obtain in these lesions, and ultra-deep protocols give the highest yield, with detection down to 0.15% diagnosing 72% of vascular malformations and 41% of complex lymphatic anomalies [172].

A molecular result changes management in a few defined circumstances and is otherwise only informative. It can select a drug: response to trametinib followed somatic *KRAS* and *MAP2K1* variants rather than germline *RASA1* and *EPHB4* [173], and the same drug salvaged a *MAP2K1*-mutant lesion after sirolimus failure [174]. It can also change how the surgical problem is understood, since orbital cavernous venous malformation is a *GJA4*-driven lesion acting through non-canonical SGK1 activation that is reversible in vitro [13,126].

A result can also trigger surveillance. Germline variants are found in a minority: 5 of 37 genotype-positive arteriovenous malformation patients carried one, four of them in *PTEN* [169]. A child treated as having fibroadipose vascular anomaly was reclassified as *PTEN* hamartoma only when a second biopsy showed a mosaic frameshift at 2.0–2.1% allele frequency [175].

**Figure 5:**
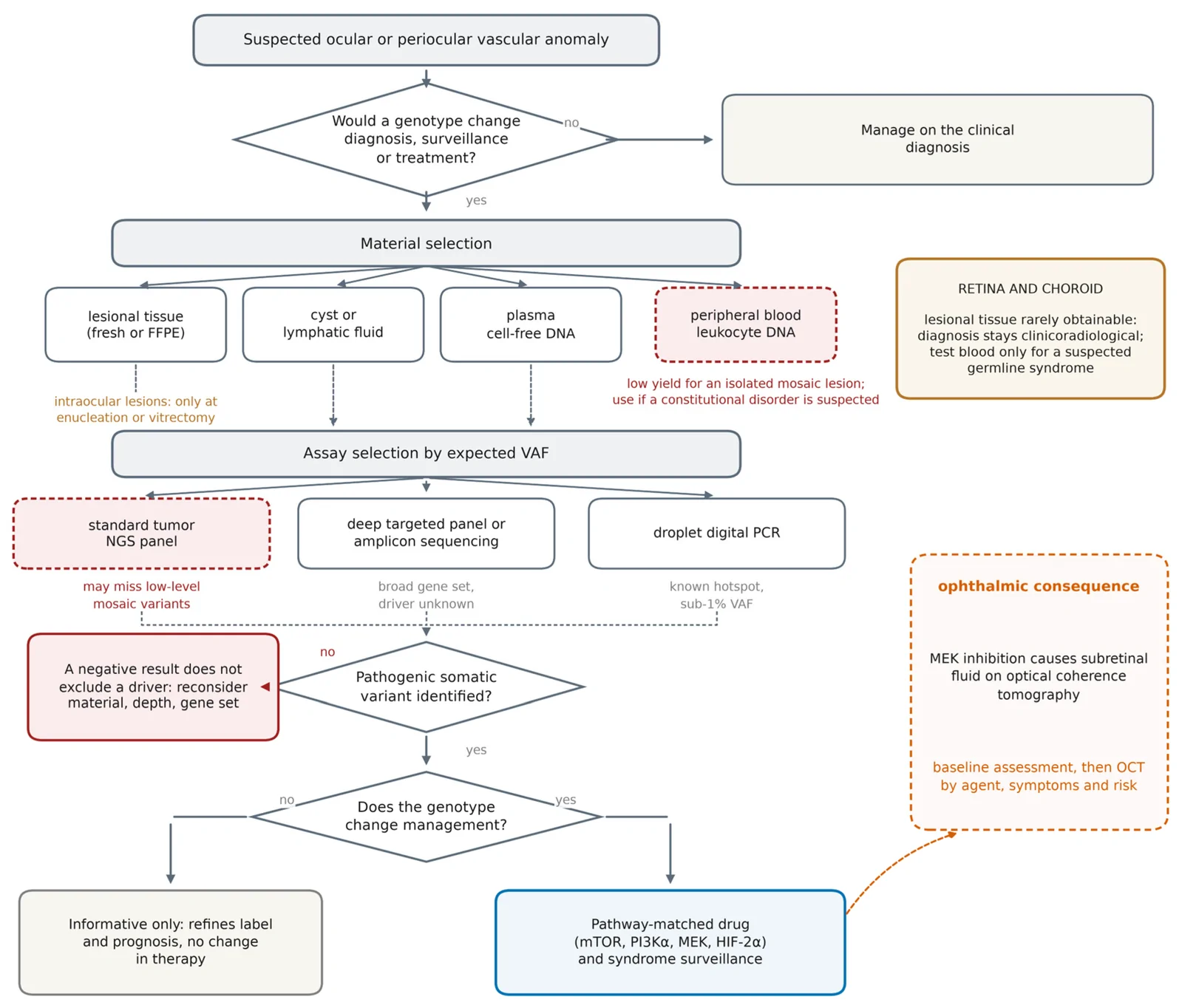
A practical framework for molecular testing and genotype-informed management of ocular and periocular vascular anomalies. Molecular diagnosis in a mosaic somatic disease is a sequence of decisions about material and assay sensitivity, and both a positive and a negative result have to be interpreted against the expected variant allele frequency. This is a framework drawn from the evidence cited below, not a guideline. The entry question is clinical rather than laboratory: testing is worth doing when a genotype would alter diagnosis, surveillance or treatment, and otherwise management proceeds on the clinical diagnosis [8,178]. Material selection is shown with three analytes suited to a somatic lesion and one that is not: lesional tissue, fresh or formalin-fixed paraffin-embedded, performs equivalently in a large mosaicism cohort [165]; cyst or lymphatic fluid is the analyte of choice for lymphatic lesions [168,172]; plasma cell-free DNA is informative for high-flow and venous lesions [168,170]; and peripheral blood leukocyte DNA gives a low yield for an isolated somatic mosaic lesion, since the driver is typically confined to lesional cells and was absent from paired blood in the defining cohorts [19,91], though blood is the appropriate material when a constitutional disorder is suspected, and cell-free DNA from blood is a separate analyte that recovers variants at ultra-deep coverage [179]. Assay selection follows the expected variant allele frequency: a conventional-depth tumor panel may miss the low-level mosaic variants characteristic of these lesions, since tissue mosaicism ranged from 0.93% to 16.53% in one high-depth cohort, with only 15 of 25 variants at or above 5% [166]; the detection floor depends on panel validation, depth and error correction rather than on a single universal threshold; deep targeted panel or amplicon sequencing is appropriate when the driver is unknown [165,167]; and droplet digital PCR recovers a known hotspot at sub-1% variant allele frequency [19,168]. The negative-result branch is marked because a negative result does not exclude a somatic driver: consensus documents treat a negative molecular result as an anticipated outcome requiring management [8,178], repeat testing of a second biopsy has changed both diagnosis and cancer surveillance in a reported case [175], and ultra-sensitive assays recover variants missed by next-generation sequencing [19]. Two exits follow the question of whether the genotype changes management, and in a large prospective cohort molecular diagnosis led to a new medical therapy in a substantial proportion of participants [172]. The adjacent panel marks the ophthalmic consequence of the drug exit: MEK inhibition produces subretinal fluid detectable on optical coherence tomography in the great majority of treated patients, mostly asymptomatic and reversible on drug cessation [181,182]. Abbreviations: FFPE, formalin-fixed paraffin-embedded; NGS, next-generation sequencing; VAF, variant allele frequency; PCR, polymerase chain reaction; OCT, optical coherence tomography; mTOR, mechanistic target of rapamycin; HIF, hypoxia-inducible factor. The panel at right states the constraint that governs the retina and choroid: lesional tissue is rarely obtainable from a seeing eye, so diagnosis stays predominantly clinicoradiological. Constitutional testing is appropriate when a germline syndrome such as von Hippel-Lindau disease is suspected, but a normal blood result cannot exclude the somatic variants that drive the *GNAQ*-associated choroidal lesions. A red dashed outline marks an option of low yield or a limitation to be weighed rather than a recommended step, and the gold box is the retina and choroid caveat.

Elsewhere the yield is smaller. The codon distinction between circumscribed and diffuse choroidal hemangioma is decisive for classification and has been proposed to explain propranolol failure in a single enucleated globe, a finding its authors call preliminary [26,40]. It does not, however, select between photodynamic therapy, radiotherapy and observation, and no series links orbital genotype to drug response [176]. Interpretation criteria are still being adapted for mosaicism [177], and graded genotype–phenotype associations vary in strength: of 45 assessed, 7 were limited and 3 without evidence [8,9]. A defined protocol for a negative result now exists [178]. Table 1 sets out material and assay selection for each clinical situation, and Figure 5 arranges the same decisions as a sequence, from the question that justifies testing to the consequence of the result.

## 12. Targeted Therapy: Mechanism, Evidence, Limitations

Grouping targeted treatments by the pathway they block shows how uneven the evidence is. Inhibition of the PI3K-AKT-mTOR pathway is the best supported axis, although what it achieves is often misunderstood. Rapamycin, an mTOR inhibitor, prevented lesion growth in mice and reduced mutant TIE2-driven AKT signaling in *TEK* p.Leu914Phe venous malformation, whereas a TIE2 kinase inhibitor did not [63]. It also normalized endothelial hyperproliferation and produced regression in vivo [183]. Table 2 ranks the pathways by how close each one is to retinal and choroidal practice, and Table 3 lists the entities with their drivers, treatment options, principal adverse effects and tier of treatment evidence.

### 12.1. HIF-2α Inhibition: The Only Prospectively Graded Retinal Endpoint

Belzutifan inhibits the terminal effector of the hypoxia axis presented in Section 9, and it is the only agent discussed here with a prospectively graded retinal endpoint, reported as a secondary endpoint of a single-arm phase 2 study. All 16 evaluable eyes in 12 participants improved on independent reading-center grading, a response rate of 100% (95% CI 79.4–100), over a median of 37.3 months [154,155]. Its regulatory position has moved since those trials: the Food and Drug Administration approved it in August 2021, and the European Medicines Agency followed in February 2025 with a conditional marketing authorization for von Hippel–Lindau disease, granted on incomplete data and carrying post-marketing obligations. The authorized indications are renal cell carcinoma, central nervous system hemangioblastoma and pancreatic neuroendocrine tumor, so treatment solely for retinal hemangioblastoma falls outside those listed authorized indications. Two observations qualify that trial result. Eight patients treated off-label for a median of 16.4 months all had stable disease, three of them a partial response, but anemia occurred in every one [184]. In a further three-patient report, belzutifan was stopped for intolerance in one patient and dose-reduced in another within a month [185]. All three received retinal photocoagulation during belzutifan treatment [185]. These cases show that local treatment may still be required in selected patients. Whether belzutifan reduces the need for laser or cryotherapy remains uncertain because comparative studies are lacking. Its hematological cost falls on patients who are otherwise well.

### 12.2. Inhibition of PI3K-AKT-mTOR

Sirolimus was tested in a phase II trial in 61 patients, which is the reference study for this pathway. Troughs were targeted at 10 to 15 ng/mL, and no complete response occurred at course 6 or 12. Of the 57 patients evaluable at course 6, 47 had a partial response, 3 were stable and 7 progressed, while marrow toxicity of grade 3 or higher occurred in 27% [186]. Sirolimus therefore holds these lesions in check, at the cost of hematological toxicity.

Sirolimus was then studied in PERFORMUS, a randomized trial in 59 children with an observational phase. Lesion volume on MRI did not differ between the treated and the observation periods, except in pure lymphatic malformations; pain, bleeding, oozing and quality of life nevertheless all improved, while oral ulcer occurred in 49.2% [187]. Symptomatic benefit is therefore better established than volume reduction.

The long-term effect on volume is only slight [188]. Severe events occurred across a wide range of blood levels rather than only at high troughs, so trough monitoring alone does not make the drug safe [189]. Topical rapamycin worked preclinically with limited systemic absorption [80], but the phase II gel trial missed its primary endpoint [190].

Orbital disease has never been tested in a randomized trial [191]. Only 10 patients have been reported, 7 with a partial and 3 with a complete response, and every complete responder had microcystic disease [192]. Volume reductions of 28.5% and 82.1% were reported in the two adults of a case report [193], with a further single patient described without a percentage [194], so the result in an individual patient cannot be predicted. A first prospective study is now under way: a phase 3 protocol in orbital lymphatic malformation assigns 30 children to sclerotherapy, to sirolimus, or to both. The assignment follows lesion complexity rather than randomization, so this design will not answer the question a randomized trial would [195].

Direct inhibition of PI3Kα, the catalytic subunit of the kinase, is supported by less evidence. Alpelisib entered clinical use on the basis of mouse models of *PIK3CA*-driven overgrowth and lymphatic disease, and of volume reduction in small refractory cohorts [164,196]. The formal response is modest and purely volumetric: a volume reduction of at least 20% at 24 weeks was reached by 12 of 32 patients (37.5%) [197].

Regulatory review of the same dataset confirmed a response in 27% (95% CI 14–44) of 37 patients [198]. Direct orbital experience now includes five patients treated with alpelisib after surgery in a seven-patient *PIK3CA*-positive series, with symptom resolution after 3–6 months; this was retrospective and uncontrolled [73]. Separately, ocular adverse events include reported panuveitis with exudative detachment and periorbital edema [199,200].

### 12.3. MEK Inhibition and Beta-Blockade

MEK inhibition is the newest axis, and all of its clinical evidence is single-arm. In a prospective pilot of 26 patients with early-stage extracranial arteriovenous malformation, trametinib gave a mean skin blanching of 53.5% and a 74% fall in peak arterial velocity [173]. Luvometinib produced an angiographic response rate of 64.3% (95% CI 35.1–87.2) in a phase 2 study of 20 adults, in which grade 3 or higher events occurred in 25% and *RASA1*-mutated patients did not respond [201].

Use in advanced disease remains limited, since only 4 of 559 drug reports in a guideline search involved trametinib [202]. Lymphatic anomalies have been treated at the case-series level only, across *ARAF*, *KRAS* and *NRAS* genotypes [125,203,204]. Pediatric dosing has not been derived for this indication but was taken from selumetinib in plexiform neurofibroma, where 70% of 50 children responded [205].

Beta-blockade is the only therapy in this field that does not depend on the genotype of the lesion, and it also has the largest randomized dataset [47]; its evidence and mechanism are presented in Section 9. Topical timolol is not equivalent to it [206]. The periocular endpoint is refraction rather than lesion volume, so size alone does not measure success [207].

### 12.4. Suppression Is Not Eradication

These drugs suppress lesions rather than eradicate them, and this limits every axis discussed above. Sirolimus improved 85% of 132 patients in the phase III VASE interim analysis, yet symptoms returned in 33 of the 61 patients (54%) who completed the two-year course, a median of 13 months after stopping, and sooner in *PIK3CA*-mutated than in *TEK*-mutated patients [176]. The same pattern appeared in a long-term cohort, in which 11 of 67 patients (16.4%) withdrew and then resumed treatment at a mean of 6.4 months [188].

Stopping can also be harmful: early discontinuation in kaposiform hemangioendothelioma was associated with rebound growth (odds ratio 3.103, 95% CI 1.529–6.299) [208]. No marker identifies the patient who can stop safely. Rebound proptosis and visual loss after withdrawal have not been described in orbital disease, although a lesion held in check by drug treatment there lies in a closed bony compartment together with the optic nerve.

### 12.5. MEK Inhibitor-Associated Retinopathy

These drugs also cause ocular adverse effects, and this is where ophthalmology adds what the dermatological and pediatric literature does not. MEK inhibitor-associated retinopathy (MEKAR) is common and mostly asymptomatic, so its reported frequency depends heavily on whether optical coherence tomography (OCT) is performed by protocol or only when the patient complains. Subretinal fluid developed in 46 of 51 patients (90%) monitored on binimetinib, yet only 9 (20%) were symptomatic; mean central retinal thickness rose from 280 to 316 μm, and the fovea was involved in 80% [181]. The incidence rises with longer exposure, to 92% on monotherapy and 100% on combination therapy [209].

Serous retinopathy occurred in 20% of patients on encorafenib plus binimetinib, against 2% on either single agent [210], although both monotherapy arms of that trial used a BRAF inhibitor, so the trial cannot isolate the MEK contribution. That attribution rests instead on binimetinib monotherapy, in which the rate reached 92%, and on pharmacovigilance data [209,211]. The onset is closely linked to the start of treatment: symptoms appear at a median of 3.5 days on binimetinib and at 15 days on cobimetinib, where the fluid resolved at 26 days [212,213].

The fluid collects at the interdigitation zone of the retinal pigment epithelium (RPE), the layer that supports the photoreceptors, and the RPE itself is dysfunctional. An abnormal Arden ratio, an electro-oculographic measure of RPE function, was found in 15 of 16 affected eyes, and anti-RPE antibodies were present in all six tested [212]. In RPE grown from induced pluripotent stem cells, selumetinib increased rod outer segment internalization and reduced aquaporin-1, a water channel, although that evidence is at preprint stage only [214].

MEK inhibitor-associated retinopathy resembles central serous chorioretinopathy on optical coherence tomography, and two features separate them. The first is the distribution of the subretinal fluid, described across 313 fluid foci in 50 eyes: it is usually bilateral (92%), multifocal (77%) and dome-shaped (73.8%), and although it reaches the fovea in most eyes (83.3%), vision recovers to baseline once the fluid clears, with no eye losing more than two Snellen lines [182]. Central serous chorioretinopathy, by contrast, is typically unilateral and unifocal. The second and more reliable feature is the choroid, which remains unchanged in thickness, area and vascularity index, whereas it thickens in central serous chorioretinopathy [215]. Subretinal fluid over a choroid of normal thickness in a patient taking a MEK inhibitor is therefore more likely to be drug-related than to represent central serous chorioretinopathy.

Because the fluid resolves, management is conservative: residual fluid persisted in only 2 of 51 patients (4%) after the drug was stopped [181]. Most patients can also continue treatment—of 655 patients receiving cobimetinib, 117 (17.9%) developed the retinopathy, fewer than 2.5% at grade 3, and 48.0% of those assessed recovered without any dose change [213]. Children and patients with pre-existing ocular disease deserve closer follow-up. Patients with pre-existing ocular disease are affected roughly twice as often (44% versus 22%) [216]. Children appear much less susceptible: a tertiary center identified only two cases of pediatric retinopathy among 104 eligible children over eight years of practice [217]; a separate cohort of 45 children yielded 3 (7%) with a new ocular finding, and those were dry eye or anterior uveitis rather than retinopathy [218]; and a further series of 26 children reported no retinopathy at all [219]. It is not known whether the adult rate applies when the indication is a vascular anomaly rather than a malignancy, since no published series in this setting has included protocolized OCT surveillance.

### 12.6. Uveitis and Retinal Vascular Occlusion

Uveitis and retinal vascular occlusion should both be mentioned when consent is taken, and each behaves differently. Uveitis is predominantly a BRAF rather than a MEK inhibitor event: pharmacovigilance data link BRAF monotherapy with anterior uveitis and MEK monotherapy with retinal and choroidal abnormalities [211]. It occurred in 4 of 65 patients (6.1%) on combination therapy, three of them Vogt–Koyanagi–Harada-like [220], with a Bayesian posterior probability of treatment-related uveitis of 5.04% (95% CrI 2.07–9.19), against an estimated 0.05% per year in the general population [221]. Any ophthalmic adverse event on BRAF inhibitors alone occurred in 1.6% of 901 patients, half of whom had uveitis, giving roughly 0.8% [222].

Retinal vascular occlusion is rare, but it threatens the continuation of treatment. Central retinal vein occlusion occurred in 3 of 546 patients across 46 prospective MEK inhibitor trials, and all three had elevated homocysteine and *MTHFR* variants [223]. Inhibition further downstream does not spare the retina either, as ERK inhibitor-associated retinopathy affected 20 of 61 patients [224].

**Table 2:** Retinal and choroidal therapeutic readiness by signaling pathway. Rows are ordered by how close the pathway is to ocular practice. Direct evidence means a study whose endpoint was measured in the retina or choroid; extrapolated evidence means the pathway was treated in another organ or another tissue. Where the direct column reads None, the therapeutic rationale is entirely inferential. Abbreviations: CI, confidence interval; OCT, optical coherence tomography. Therapeutic readiness is a qualitative judgment based on lesion-specific benefit and risk, anatomical applicability, clinical experience and regulatory context; it is not assigned automatically from a study-design tier. “Actionable now” denotes an established treatment or a clinically available option for selected patients; “pathway actionable” denotes clinical evidence in another setting; “investigational” denotes preliminary lesion-specific use; “preclinical target” requires an experimental therapeutic result. “No direct evidence” denotes an untested lesion–intervention pairing. Evidence tiers and their anatomical scope are defined in Table 3.

Pathway/Gene	Ocular Lesion	Agent	Direct Retinal or Choroidal Evidence	Extrapolated Evidence	Evidence Level	Therapeutic Readiness
VHL-HIF-2α/*VHL*	Retinal hemangioblastoma	Belzutifan	16 of 16 evaluable eyes improved, 100% (95% CI 79.4–100), median 37.3 months; secondary endpoint of a single-arm phase 2 study [154,155]	Not required	Phase 2, single-arm, prospectively graded	Clinical option for selected patients; retinal-only use is outside the listed authorized indications. Anemia in 90%; local treatment may still be required in selected patients [154,184,185]
PI3K-AKT-mTOR/*PIK3CA*, *TEK*	Orbital lymphatic malformation	Sirolimus, alpelisib	None. Direct molecular evidence concerns orbital lymphatic and lymphaticovenous malformations [72,73], not retinal racemose malformation	Phase II sirolimus in complicated vascular anomalies, *n* = 61 [186]; alpelisib real-world 37.5% (12/32) [197] and in *TEK*-mutated venous malformation, *n* = 3 [225]; miransertib phase 1/2 safety population *n* = 49, of whom 45 had PROS and none had ophthalmic surveillance reported [226]; phase 3 orbital lymphatic protocol recruiting [195]; orbital alpelisib case series, *n* = 5 treated [73]	Phase II/III in non-ocular disease; retrospective orbital treatment reports; no direct retinal endpoint	Pathway actionable outside the eye; orbital use investigational; no direct retinal efficacy evidence
RAS-MAPK-ERK/*KRAS*, *MAP2K1*	Extraocular and periocular arteriovenous malformation	Trametinib, luvometinib	None as therapy. The retina appears only as toxicity: subretinal fluid in 46 of 51 patients (90%) under protocol OCT [181]	Single-arm pilot *n* = 26 [173]; phase 2 *n* = 20 [201]	Phase 2, single-arm, non-ocular indication	Pathway actionable, but the eye is the toxicity organ; OCT before and during treatment
Gq/11/*GNAQ*, *GNA11*	Diffuse and circumscribed choroidal hemangioma; Sturge–Weber	Topical rapamycin, laser; MEK inhibition preclinical	None. No choroidal or glaucoma endpoint has been reported	Cutaneous port-wine stain, topical rapamycin or sirolimus with pulsed dye laser in small randomized trials with inconsistent endpoints [227,228]; uncontrolled sirolimus in Sturge–Weber syndrome with neurological endpoints [229]; MEK inhibition corrects the barrier defect in p.R183Q endothelium in vitro [32]	Preclinical plus cutaneous randomized data	Experimental Gq-pathway rationale; cutaneous clinical evidence does not establish choroidal efficacy
Junctional/*GJA4*	Orbital cavernous venous malformation	SGK1 inhibition	None	Cell and mouse work in cutaneous, hepatic and intracranial *GJA4* lesions [126,137]	Preclinical only	Preclinical target; the clearest driver-defined disease in this set [13]
CCM, MEKK3-KLF2/4/*KRIT1*, *MAP3K3*	Retinal cavernous lesion	Propranolol	None. The retinal lesion has never been genotyped [146]	Randomized open-label blinded-endpoint phase 2 pilot in familial cerebral cavernous malformation, *n* = 83, intracranial endpoints only [230]	Phase 2 in intracranial disease	Unknown ocular biology
Adrenergic/no recurrent driver	Periocular infantile hemangioma	Propranolol, timolol	Astigmatism as the periocular endpoint [207]	Randomized phase 2–3, 60% versus 4%, no periocular subgroup [47]	Randomized, but not in a periocular subgroup	Actionable now, and the counterexample: effective without a known driver

**Table 3:** Ocular vascular entities, their drivers, treatment options, key adverse effects and level of treatment evidence. Design-based tiers: tier 1, prospective study with a graded ocular endpoint; tier 2, randomized or prospective study in non-ocular vascular disease; tier 3, retrospective series or case reports; tier 4, experimental therapeutic evidence only. These categories describe study design, not comparative certainty, regulatory approval or treatment recommendations. Each tier applies to the specified intervention and anatomical setting. “No direct evidence” means that the relevant lesion–intervention pairing has not been tested and is distinct from tier 4. Established phenotype-directed treatments are identified separately. Molecular associations and treatment evidence should not be conflated. The arrow denotes a mechanistic consequence, as in VHL loss → HIF-2α stabilization.

Ocular Entity	Driver/Pathway	Molecular Evidence	Treatment Options	Key Adverse Effects, Including Ocular Toxicity	Treatment Evidence: Tier and Anatomical Scope
Retinal capillary hemangioblastoma (VHL disease)	VHL loss → HIF-2α stabilization	Established mechanism; genetic diagnosis routine [148,149]	Belzutifan (systemic); laser/cryotherapy remain local standard [154,185]	Anemia ~90%, hypoxia, fatigue (systemic); no direct ocular toxicity reported [154]	1—single-arm phase 2, 16/16 evaluable eyes in 12 participants improved, no comparator [154,155]
Circumscribed choroidal hemangioma	*GNAQ* codon 209 (allele distribution unsettled)	Recurrent driver in ≥2 cohorts (28/33; 6/6) [40,41]	None pathway-directed; PDT, photocoagulation, TTT, radiotherapy, anti-VEGF [54,55]	PDT/laser destructive by design; anti-VEGF minimal [54]	No direct evidence for pathway therapy in this lesion
Diffuse choroidal hemangioma (Sturge–Weber)	*GNAQ* codon 183	Limited direct ocular sequencing; supported by cutaneous findings [26,40]	None; topical rapamycin/sirolimus trialed in skin only [190,228]	mTOR class: oral ulceration; no ocular endpoint data [187]	No direct ocular pathway-therapy evidence; tier 2 for cutaneous trials [228]
Sturge–Weber glaucoma/ocular SWS	*GNAQ* (±*GNA11*), mosaic	Strong for skin/brain; limited ocular tissue data [49]	None ocular; topical rapamycin or sirolimus tested in cutaneous port-wine stain only [190,228]	Systemic mTOR class effects [187]	No direct evidence for ocular pathway inhibition; a single preliminary propranolol report is not evidence for a targeted regimen [26]
Retinal racemose malformation	Not established	No direct lesional genotyping in the cited studies; orbital findings in CVMS do not establish a retinal driver [72]	No established pathway-directed therapy	Not applicable; no established pathway-directed regimen	No direct evidence for pathway-targeted treatment of this lesion
Non-cavernous venous malformation with periocular/orbital involvement	*TEK* or *PIK3CA* in general VM cohorts; distinct from *GJA4*-associated cavernous venous malformation	Drivers established in general VM cohorts; these detection frequencies are not specific to periocular/orbital lesions [58,59,65]	Sirolimus; alpelisib (incl. TIE2-mutated VM, preliminary) [187,197]	mTOR/PI3K class effects [187]	Tier 2 for sirolimus and tier 3 for alpelisib in the cited non-ocular cohorts; orbital application is extrapolated [187,197]
Orbital lymphatic malformation	*PIK3CA*	Direct orbital *PIK3CA* evidence [72,73]; larger detection cohorts remain predominantly non-orbital [68]	Sirolimus [192,193,194]; alpelisib, five orbital cases [73]	mTOR/PI3K class effects [187]	Tier 3 for orbital treatment; tier 2 for non-orbital sirolimus [73,187,192,193,194]
Periocular/extraocular arteriovenous malformation	*KRAS*, *MAP2K1*, *BRAF* (RAS/MAPK)	Recurrent drivers in extraocular AVM cohorts; periocular/orbital molecular evidence remains limited [85,86,87]	Trametinib, luvometinib (non-ocular pilots) [173,201]	MEK-inhibitor retinopathy, uveitis, retinal vein occlusion; OCT monitoring recommended [181,223]	Tier 2 for non-ocular AVM; no direct ocular efficacy study [173,201]
Orbital cavernous venous malformation	*GJA4* p.Gly41Cys (junctional)	Two cohorts (25/26; 44/59); cavernous-phenotype-specific [13,14]	No established targeted therapy; SGK1-directed experimental rationale from non-orbital *GJA4* models [126,137]	Not applicable	Tier 4 in non-orbital *GJA4* models; no direct orbital therapeutic study [126,137]
Periocular infantile hemangioma	No somatic driver identified	No recurrent somatic driver established; the cited mechanistic studies did not assay Gq/11 genes [15,16]	Propranolol (standard of care, non-targeted) [47]	Systemic beta-blocker class effects (hypoglycemia, bronchospasm), infrequent and no more common than placebo in the pivotal trial [47]; no ocular toxicity reported	Established phenotype-directed treatment; pivotal randomized evidence is not specific to a periocular subgroup [47]
Pyogenic granuloma (ocular adnexa)	Ocular driver unresolved; *BRAF*/RAS-MAPK associations derive from non-periocular lesions	*BRAF* p.Val600Glu in 8 of 10 port-wine-stain-associated lesions and 3 of 25 sporadic; no *BRAF* p.Val600Glu in 28 periocular specimens; RAS genes never sequenced periocularly [120,121,122]	Excision standard; MEK/BRAF inhibition theoretical	MEK class retinopathy if used systemically [223]	No direct evidence for pathway therapy in periocular lesions
Congenital hemangioma (RICH/NICH)	*GNAQ*/*GNA11* codon 209	Recurrent driver [10,11]	None; observation or surgery	Not applicable	No direct evidence for pathway-targeted treatment
Cerebral cavernous malformation (ocular counterpart unproven)	CCM complex (KRIT1, CCM2, PDCD10)	Established in CNS; ocular counterpart unresolved [144,145,146]	Propranolol trialed in familial CCM (CNS) [230]	—	Tier 2 in CNS disease; no direct retinal therapeutic evidence [230]

## 13. Future Directions

### 13.1. Disease Models

Disease models are the most achievable near-term advance. Patient-derived vascular organoids grown from a patient’s own cells reproduce a capillary malformation phenotype [231]; a venous endothelial platform carrying a knocked-in *TEK* p.Leu914Phe allele has been screened against many existing drugs, and the screen identified a candidate for repurposing [232]; and a zebrafish *NRAS* p.Gln61Arg model works both as a disease model and as a screening tool [233].

None of these models derives from an orbital or adnexal lesion, and none exists for *GJA4*-driven cavernous venous malformation, the most common orbital vascular lesion [13]. Building such a model would therefore be the most useful contribution this specialty could make. The proliferative index of that lesion is near zero, so it remains unclear whether a drug directed at growth could act on it at all [46].

### 13.2. Correcting or Silencing the Driver

Correction of a somatic driver at the DNA level, including base or prime editing, has not been established as a treatment for ocular vascular malformations. No report of such DNA correction in a vascular-malformation model was identified in the focused supplementary search described in Section 2. RNA-directed approaches must be considered separately: Saito et al. used CRISPR/CasRx to reduce mutant *KRAS* expression and suppress brain arteriovenous malformation development in mice [234]. This is preclinical RNA knockdown, not correction of the DNA variant, and does not establish efficacy in ocular disease. No antisense or siRNA therapeutic report directed against *PIK3CA*, *TEK* or *KRAS* in a vascular anomaly was identified in that focused search; experimental knockdown of other pathway components was not counted as a driver-directed treatment.

### 13.3. Getting the Drug to the Posterior Segment

The route of administration is the constraint this field has not yet addressed. Every agent discussed here is given systemically for a lesion confined to one eye, and this mismatch has a cost: belzutifan shrinks the retinal hemangioblastoma but causes anemia in most patients treated [184], and it is discontinued or dose-reduced for intolerance in a fraction of them [185]. Local delivery would separate the ocular benefit from the systemic dose, and a precedent exists in adjacent practice. Intravitreal anti-VEGF reduces the vascularity and size of retinal capillary hemangioblastoma on quantitative fundus analysis [235], and it is given before and after photodynamic therapy for juxtapapillary lesions [236]. Topical sirolimus with pulsed dye laser has been tested against placebo in cutaneous port-wine stain [228], which shows that an mTOR inhibitor can reach lesional endothelium without a systemic dose. What is missing is the same reasoning applied to a pathway inhibitor in the posterior segment: no intravitreal, suprachoroidal or sustained-release formulation of an mTOR, MEK or HIF-2α inhibitor has been tested in an ocular vascular anomaly. The posterior segment is where these formulations are hardest to deliver and where the endpoint is easiest to measure, so the gap is one of trial design rather than of pharmacology.

### 13.4. Target Engagement and Response Biomarkers

Proof of principle at the target level exists. Gαq is the signaling protein that the mutation leaves switched on, and both knockdown of PLCB3 and direct inhibition of Gαq reduce the angiopoietin-2 output of *GNAQ* p.Arg183Gln endothelium [28]. Angiopoietin-2 is also the only validated response biomarker here, and it is validated only in lesions that overproduce it.

Serum angiopoietin-2 separates kaposiform lymphangiomatosis and kaposiform hemangioendothelioma with coagulopathy from generalized lymphatic anomaly, and it falls when a lesion responds to sirolimus [79]. Because it also reflects Gq signaling in capillary malformation endothelium [28], it is worth testing in the Sturge–Weber eye. No marker of disease activity exists for the periocular lesions themselves, and it is unknown whether VAF predicts the size or the durability of response [173,176].

### 13.5. Registries and Surveillance

The registry problem is specific and can be corrected. The multi-institutional cohorts and registry series that generate this field’s genotype–phenotype and treatment data record lesion subtype and driver gene, but none of them reports its cases by anatomical site, so orbital and periocular patients cannot be identified [172,176,188]. The prevalence, genotype spectrum and drug response of periocular disease therefore cannot be determined.

Two changes would be simple to introduce: an orbital and adnexal field in those registries, and OCT by protocol in every patient started on a MEK inhibitor. Together they would close two of the largest gaps identified in this review.

## 14. Conclusions

Recent molecular studies have substantially changed our understanding of vascular lesions of the eye, orbit and periocular tissues. Many entities that were historically grouped by appearance or histology are now known to arise from distinct somatic alterations, while lesions with different clinical names may share the same molecular pathway. The traditional phenotype-based classification therefore remains useful in clinical practice but increasingly needs to be interpreted alongside genotype. In particular, the Gq/11, PI3K/AKT/mTOR–TIE2, RAS/MAPK, junctional *GJA4*/CCM and VHL/HIF pathways account for a substantial proportion of the lesions discussed in this review. The strength of genotype–phenotype correlation is itself lesion-dependent, ranging from driver-defined disease (the orbital cavernous venous malformation) through limited direct orbital evidence (*PIK3CA*-associated lymphatic malformations) to conditions whose driver remains unresolved in the cited studies (retinal racemose malformation and infantile hemangioma); the tiers of treatment evidence defined in Table 3 should be read alongside every therapeutic statement in this review.

Genotype alone, however, does not fully determine phenotype. Developmental timing, the affected cellular lineage and the functional effect of a particular variant all appear to contribute to the final clinical presentation. This is particularly well illustrated by *GNAQ*-associated disease, in which different variants and cellular contexts are associated with capillary malformations, choroidal hemangiomas and melanocytic neoplasia. Such observations explain why morphology cannot always predict the underlying molecular abnormality and why closely related lesions may differ in natural history and treatment response.

The clinical translation of these findings remains uneven. The strongest direct ocular evidence is currently available for HIF-2α inhibition with belzutifan in VHL-associated retinal hemangioblastoma. Propranolol is well established for infantile hemangioma, although no recurrent somatic driver has been identified for this lesion. PI3K, mTOR and MEK inhibitors have become important treatments for selected vascular anomalies elsewhere in the body, but ophthalmic experience remains limited and is based largely on extrapolation, small series or individual cases. Gq/11- and *GJA4*-directed treatment remains preclinical. The potential benefit of systemic pathway inhibition must also be considered together with treatment-related ocular toxicity, particularly MEK inhibitor-associated retinopathy.

Several clinically relevant gaps remain. Orbital varix, conjunctival vascular and lymphatic malformations and isolated retinal arteriovenous malformation still lack adequate molecular characterization. For orbital lymphatic disease, early molecular and treatment evidence is now available [72,73], but broader cohorts are needed. Prospective ophthalmic assessment of patients receiving targeted therapies, including protocolized OCT during MEK inhibition, would provide information that is currently missing. Better anatomical reporting in vascular-anomaly registries would also allow orbital and periocular disease to be studied as distinct groups. For ophthalmologists, the practical value of molecular classification is therefore not to replace clinical diagnosis, but to refine it and, where sufficient evidence exists, to connect an individual lesion with a more specific prognosis, diagnostic strategy or treatment.

## Figures and Tables

**Table 1:** Material and assay selection for molecular diagnosis of periocular and orbital vascular anomalies, with reported analytical detection limits separated from observed variant allele frequencies (VAFs) and diagnostic yields. Detection limits apply to the cited assays and should not be inferred from the lowest observed VAF or the proportion of positive samples. FFPE, formalin-fixed paraffin-embedded.

Material	Assay	Reported Analytical Detection Limit (VAF)	Observed VAF or Diagnostic Yield	Good for	Limitation
Blood leukocytes	Deep panel	Sub-1% with PNA-clamp ddPCR [19]	Poor yield as primary material for somatic testing [165]	Germline comparator; germline *PTEN* [169]	Negative result uninformative [165]
Tissue, fresh or FFPE	Deep panel, approximately 2000× [165]	Approximately 1% for the cited panel [166]	80.8% diagnostic yield [166]	First line; FFPE equals fresh [165]	Dilutes endothelial variants [71]
Endothelium-enriched	Sorting, laser capture	Not reported	Observed VAF: 5% in bulk tissue versus 31% in endothelium [88]; 21.1% in choroidal vessels [26]	Small orbital, choroidal specimens [70]	Culture selects *PIK3CA* [165]
Cyst fluid	Multiplex ddPCR	Not reported	7 of 7 positive [168]	Macrocystic lymphatic malformation [168]	Hotspots only [168]
Plasma cell-free DNA	ddPCR; barcoded panel	0.05% with the cited assay at 104,000× [179]	Variable by lesion and specimen [168,179,180]	Fast-flow, venous; effusion [180]	Insensitive in lymphatic lesions [168]
Draining-vein blood	Deep cell-free DNA	Not reported	8 of 10 positive [170]	AVM if biopsy risks bleeding [171]	VAF falls with distance [170]
Lymphatic fluid	Ultra-deep sequencing	0.15% with the cited protocol [172]	Diagnostic yield: 72% of vascular malformations and 41% of complex lymphatic anomalies [172]	Complex lymphatic anomaly [172]	Yield class-dependent [172]

## Data Availability

No new data were created or analyzed in this study. Data sharing is not applicable to this article.

## Abbreviations

The following abbreviations are used in this manuscript:

AVM	arteriovenous malformation
CCM	cerebral cavernous malformation
CLOVES	congenital lipomatous overgrowth, vascular malformations, epidermal nevi and skeletal anomalies
CM	capillary malformation
CM-AVM	capillary malformation-arteriovenous malformation syndrome
ddPCR	droplet digital polymerase chain reaction
ERK	extracellular signal-regulated kinase
FFPE	formalin-fixed paraffin-embedded
GLUT1	glucose transporter 1
HIF	hypoxia-inducible factor
ISSVA	International Society for the Study of Vascular Anomalies
MEK	mitogen-activated protein kinase kinase
MEKAR	MEK inhibitor-associated retinopathy
mTOR	mechanistic target of rapamycin
NGS	next-generation sequencing
OCT	optical coherence tomography
PROS	*PIK3CA*-related overgrowth spectrum
RPE	retinal pigment epithelium
VAF	variant allele frequency
VEGF	vascular endothelial growth factor
VHL	von Hippel–Lindau
